# The PainSMART project: Protocol for a research program on effectiveness, mechanisms of effect and patient-practitioner experiences of the PainSMART-strategy as an adjunct to usual primary care physiotherapy management for musculoskeletal pain

**DOI:** 10.1371/journal.pone.0316806

**Published:** 2025-01-30

**Authors:** Richard Thompson, Maria Fors, Ann-Sofi Kammerlind, Pia Tingström, Allan Abbott, Kajsa Johansson

**Affiliations:** 1 Unit of Physiotherapy, Division of Prevention, Rehabilitation and Community Medicine, Department of Health, Medicine and Caring Sciences, Linköping University, Linköping, Sweden; 2 Rehab Finspång, Region Östergötland, Finspång, Sweden; 3 Department of Activity and Health and Department of Health, Medicine and Caring Sciences, Linköping University, Linköping, Sweden; 4 Futurum, Region Jönköping County, Jönköping, Sweden; 5 Division of Nursing Sciences and Reproductive Health, Department of Medical and Health Sciences, Linkoping University, Linkoping, Sweden; 6 Department of Orthopaedics, Linköping University Hospital, Linköping, Sweden; PLOS, UNITED KINGDOM OF GREAT BRITAIN AND NORTHERN IRELAND

## Abstract

**Background:**

Musculoskeletal pain (MSKP) disorders entail a significant burden for individuals and healthcare systems. The PainSMART-strategy has been developed aiming to reduce divergences between patients and healthcare practitioners in their understanding of MSKP by providing a shared basis for communication and to facilitate patients’ self-management of MSKP. The objective of the PainSMART-project is to evaluate the effects of the PainSMART-strategy as an adjunct to usual physiotherapy management compared to usual physiotherapy management alone.

**Methods:**

The PainSMART-project is a research program with a collective suite of studies utilising mixed methods, centred around a randomised controlled trial (ClinicalTrials.gov NCT06187428). Subjects: Adults (18 years or older) seeking primary care for MSKP who are triaged and booked for an initial physiotherapy consultation at five primary care physiotherapy departments within the Swedish public healthcare regions of Östergötland and Jönköping. A total of 490 subjects will be randomised to receive one of two possible interventions.

**Interventions:**

Both groups will receive usual physiotherapy management for benign MSKP. The intervention group will also receive the PainSMART-strategy consisting of an educational film, reflection and reinforcement of the film’s key messages prior to the initial physiotherapy consultation and a patient-practitioner discussion based on the film.

**Outcome:**

The primary outcome is 1) between group mean change over time from baseline to 24 hours post initial physiotherapy consultation and baseline to 3 months regarding self-reported average pain intensity and pain self-efficacy. Secondary outcomes include similar measurements for MSKP illness perception, reassurance of benign nature, pain coping, physical activity, analgesic medication use, sick leave, healthcare use and direct healthcare costs. Physiotherapist and patient reported experience measures and qualitative evaluation of the effects of the PainSMART-strategy on communication at the initial physiotherapy consultation will also be explored.

**Discussion:**

This study will investigate potential added effects of PainSMART-strategy upon usual primary care physiotherapy for MSKP.

## Introduction

Musculoskeletal disorders are a major health problem and entail a significant burden for individuals and healthcare systems [1]. In Sweden, musculoskeletal disorders, in particular spinal pain, are one of the leading causes of disability and their burden is increasing [2]. As of February 2023, around 33,000 people in Sweden were unfit to work due to musculoskeletal disorders [3]. Approximately 30,000 unique individuals visit primary care physiotherapy departments within the Östergötland healthcare region (population 450 000) for musculoskeletal disorders each year [4]. High-value musculoskeletal healthcare is therefore imperative for individuals, healthcare systems and society at large [5]. With an increasing demand for services there is a need to move towards new ways of managing musculoskeletal pain (MSKP). The ‘Nära Vård’ (Close care) initiative in Sweden has been developed with the aim of creating a more patient-centered, accessible healthcare system where practitioners and patients share responsibility for managing the patient’s health [6]. Part of this initiative involves the evolution of healthcare pathways [6].

Managing MSKP is complex and depends on an array of biopsychosocial factors. Improving MSKP care requires a paradigm shift in the understanding of pain and an increased focus on helping people self-manage MSKP episodes [7, 8]. Effective early management of MSKP is critical as prolonged activity in the nervous system can drive neuroplastic changes that make pain more difficult to treat [7–11]. According to the Common-sense model of self-regulation (CSM), how an individual’s MSKP progresses over time depends on how they perceive their MSKP, what coping strategies they adopt and how they manage MSKP [12]. A significant body of evidence supports the CSM and indicates that more negative MSKP illness representations are associated with increased pain intensity and poorer physical function [13, 14]. The influence of MSKP illness perceptions is further highlighted by modern pain theories, such as the predictive processing theory, that consider pain the product of an individual’s perception of the potential threat to bodily integrity [9, 11]. As such, an individual’s MSKP experience is regulated by the meaning, perceived causes and consequences they assign to their MSKP [9–11, 15]. For example, it is common amongst the general public and even some healthcare professionals, for the body to be likened to a machine and MSKP considered a sign of damage [7, 16, 17]. Such misconceptions have been reported to lead to increased pain intensity, disability, use of passive coping-strategies, over-medicalisation and an overuse of imaging and surgical interventions that are often iatrogenic [7, 14, 18]. Other psychological factors, such as pain self-efficacy or psychological flexibility, further influence an individual’s management of MSKP and affect prognosis [7, 19]. For instance, pain self-efficacy, defined as the perception of one’s ability to carry out activities when in pain, has been found to link pain to disability, whilst higher pain self-efficacy is thought to be protective of the development of chronic MSKP [19–21]. Psychological flexibility has been found to be significantly associated with physical function whilst fear of movement, fear of pain and avoidant or passive coping strategies are risk factors for the development of chronic pain [7, 15, 22–25]. Collectively, this literature highlights why MSKP illness perceptions and psychological factors, are considered important targets for MSKP interventions.

Educational interventions have the potential to improve outcomes for people with MSKP by targeting factors such as MSKP illness perceptions and pain self-efficacy. However, more evidence is required to establish the most effective educational interventions. The need for improved MSKP educational materials was highlighted in 2020 when The Lancet published a list of ten recommendations to improve care of low back pain (LBP) [18]. Six of these recommendations stated the need for improved educational and self-care support materials and the need to scientifically evaluate such materials [18]. Previous evidence has shown that online and in-person MSKP educational interventions can have positive effects on pain intensity and disability [26–28]. However, these studies have only included chronic pain populations, been limited to LBP or used educational interventions based on outdated pain theories and not developed in collaboration with people with MSKP [26–29]. National and international guidelines already recommend pain education as standard practice for acute and chronic MSKP, but guideline uptake has been poor [23, 24, 30, 31]. A meta-review found that barriers to the implementation of guidelines, such as providing MSKP education, are a lack of time for professionals to keep up to date with research and to communicate research-based guidelines to patients [32]. A more efficient MKSP management pathway may therefore be facilitated by the development of educational interventions that are concise, easily administered and delivered directly to people with MSKP [32].

Communication between patients with MSKP and healthcare practitioners can be difficult and may be hampered by divergences in understanding and expectations [33]. For example, qualitative evidence consistently finds that people with MSKP want definitive diagnoses and an explanation of the cause of their pain [16, 34, 35]. However, as MSKP is considered an emergent neurophysiological phenomenon, explaining it can be complex and providing a definitive diagnosis is often impossible [8, 11]. A divergence in expectations can therefore arise in a clinical consultation when healthcare practitioners approach MSKP as a complex emergent phenomenon and a patient views MSKP through a traditional biomedical lens. Indeed, a patient’s lack of knowledge or uncertainty about their condition has been shown to hinder effective consultations and the implementation of evidence-based healthcare [32, 33]. The PainSMART-strategy has been developed in part to address such divergences in understanding by administering an educational intervention prior to a healthcare consultation. This to help reduce divergence in understanding between a patient and healthcare practitioner by providing a shared basis for communication. It is hoped that the implementation of the PainSMART-strategy may facilitate patient-practitioner interaction around MSKP and result in improved patient outcomes compared to usual healthcare management.

## Aims and objectives

The PainSMART-project is a research program with a collective suite of sub-studies aiming to investigate the effectiveness of the PainSMART-strategy, factors associated with outcomes and explore patients and physiotherapists experiences. The objective of the PainSMART-project is to evaluate administering the PainSMART-strategy as an adjunct to usual physiotherapy management compared to usual physiotherapy management alone.

Hypotheses for confirmatory research questions:

Sub-study 1) Exposure to the PainSMART-strategy as an adjunct to usual physiotherapy management improves the following patient reported outcomes significantly more than usual physiotherapy management alone for patients with MSKP

- Reduction in pain intensity.
- Higher pain self-efficacy.
- Lower MSKP illness perceptions.
- Higher levels of reassurance of the benign nature of MSKP.
- More adaptive MSKP coping and psychological flexibility.
- Higher self-reported levels of physical activity.
- More positive global ratings of change.

Sub-study 2) Exposure to the PainSMART-strategy as an adjunct to usual physiotherapy management improves the following process outcomes significantly more than usual physiotherapy management alone for patients with MSKP

- Lower number of healthcare visits, referrals for diagnostic imaging and to specialist/tertiary care for MSKP, lower analgesic medication use, fewer sick leave days and lower direct costs.

Sub-study 3) Exposure to the PainSMART-strategy as an adjunct to usual physiotherapy management improves the following patient and practitioner reported experience measures significantly more than usual physiotherapy management alone for patients with MSKP

- More positive and concordant patient and physiotherapist evaluations of MSKP-related shared understanding, communication, participation, involvement and emotional support at the initial physiotherapy consultation.

Sub-study 4) Improvements in MSKP illness perceptions and higher levels of reassurance of the benign nature of MSKP mediate improved pain intensity and pain self-efficacy as a result of exposure to the PainSMART-strategy compared to usual physiotherapy management alone.

Exploratory research questions:

Sub-study 5) What baseline factors are predictive of improved patient outcomes after exposure to the PainSMART-strategy?

Sub-study 6) What baseline factors are predictive of the persistence of MSKP?

Sub-study 7) What types of specific coping strategies are associated with patient outcomes after exposure to the PainSMART-strategy?

Sub-study 8) Is pain self-efficacy a potential mediator of the PainSMART-strategy´s effect on health outcomes?

Sub-study 9) What are patients and physiotherapists experiences of the PainSMART-strategy?

Primary research question for the qualitative part of the research program:

Sub-study 10) Does exposure to the PainSMART-strategy influence communication around pain at the initial physiotherapy consultation?

Dimensions to explore regarding the qualitative part of the research program:

1. Describe how physiotherapists and patients incorporate the PainSMART-strategy during the initial physiotherapy consultation.
2. Explore potential explanations for the quantitative results of the randomized controlled trial (RCT).
3. Describe the participating patients’ understanding of MSKP and explore how this may relate to the PainSMART-strategy’s theoretical underpinnings.
4. Explore if patients’ understanding of MSKP diverges from the understanding of the physiotherapist during the initial consultation.

## Methods

### Trial design

The PainSMART-project is a research program with a collective suite of 10 sub-studies utilising mixed methods, centred around a randomised, control group blinded, superiority trial with two parallel groups. A 1:1 group allocation ratio will be applied. The control group will be blinded. One independent statistician will be responsible for database development and linkage of patient and physiotherapist data. Another independent statistician will be blinded for the primary analyses. The study protocol follows the standard protocol item recommendations for intervention trials (SPIRIT) checklist provided in S1 File. The study protocol has been prospectively registered in ClinicalTrials.gov (NCT06187428) and the World Health Organisation (WHO) Trial Registration Data Set is presented in S2 File. The current study protocol version is V1.2 as of 25/09/2024. The research program has also received competitive peer reviewed grant funding through the Medical Research Council of Southeast Sweden (FORSS-963776, 981776 & 995094) and Region Östergötland (RÖ-990984 & 987927). The results of the RCT will be reported according to the Consolidated Standards of Reporting Trials (CONSORT) statement and the CONSORT patient-reported outcomes checklist [36]. The qualitative data collection will be reported in line with the Consolidated Criteria for Reporting of Qualitative research (COREQ) [37].

### Study setting

This study is a multi-centre RCT that will be conducted at five primary care physiotherapy departments within the Swedish regions of Östergötland (RÖ) and Jönköping (RJL). All four physiotherapy departments within RÖ and one physiotherapy department within RJL have agreed to participate. Collectively these centres employ around 130 physiotherapists and provide initial consultations to approximately 30 000 unique individuals in a one-year period. A list of the participating physiotherapy departments is available from the corresponding author on request.

### Eligibility criteria

The eligibility of potential participants in the study will be based the following criteria:

Inclusion criteria

- Patients who, via telephone or online text-based triage, are judged to have benign MSKP and are booked for an initial physiotherapy consultation.
- Adult patients (18 years or older).

Exclusion criteria:

- Patients who are judged to require urgent medical examination due to suspected serious pathology (red flags).
- Patients who are booked for an initial physiotherapy consultation on the same day as, or the day directly following triage.
- Patients referred for physiotherapy following consultation with a tertiary care practitioner (e.g. orthopaedic surgeon, rheumatologist, neurologist).
- Patients who cannot communicate in Swedish to the equivalent of a 12-year-old native speaker (as judged by the triaging physiotherapist).
- Patients who, through visual impairments, are unable to complete the necessary questionnaires for the study.
- Patients who are booked for an initial consultation with a physiotherapist who has not consented to taking part in the study.

**Fig 1:**
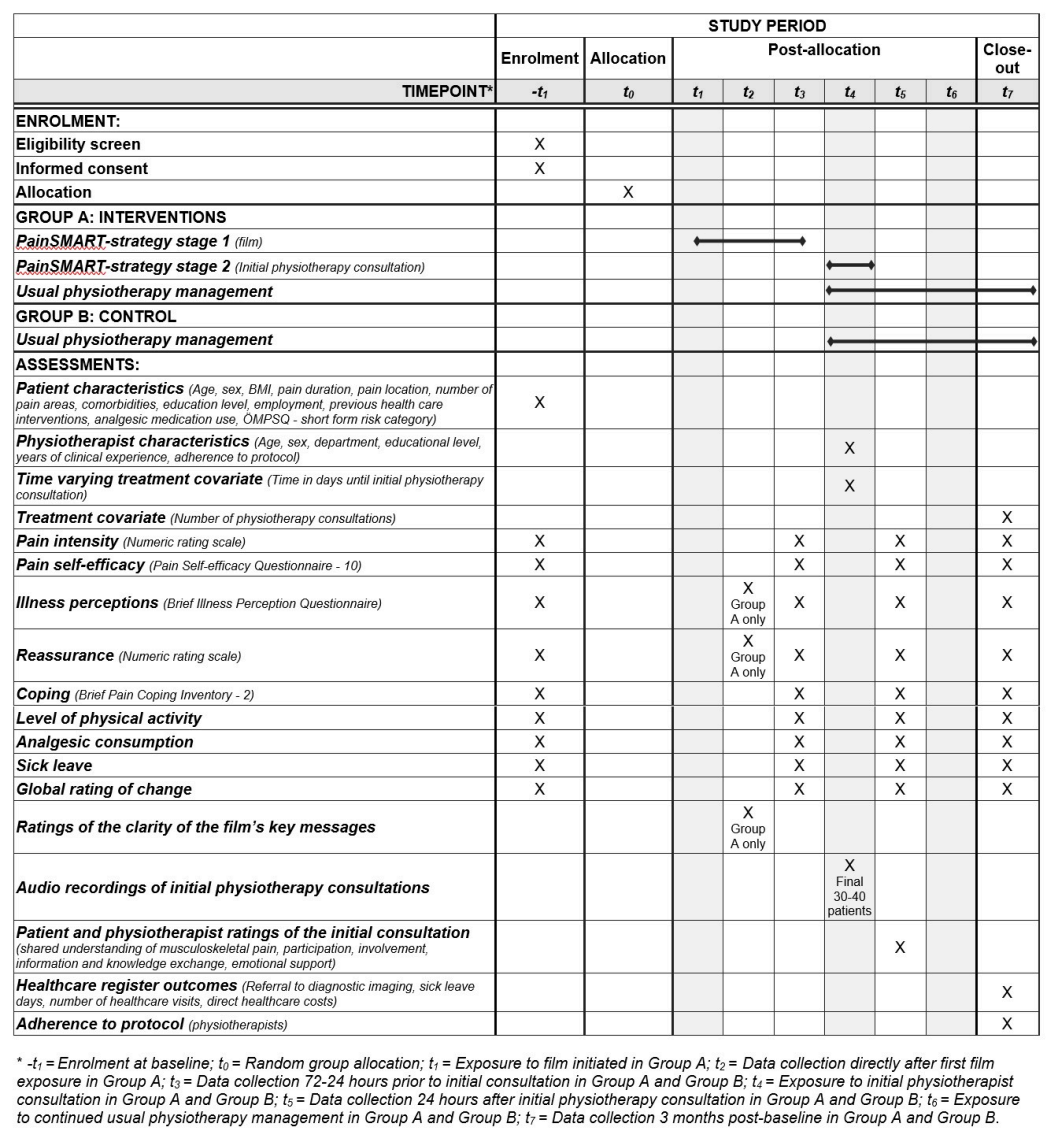
SPIRIT schedule of enrolment, interventions, and assessments.

SPIRIT schedule of enrolment, interventions, and assessments through the study is shown in Fig 1. All physiotherapists who provide care to patients booked for a consultation for MSKP at one of the participating physiotherapy departments are eligible to participate.

### Intervention description and rationale

The intervention in this study is the PainSMART-strategy as an adjunct to usual physiotherapy care and is described according to the Template for Intervention Description and Replication (TIDieR) checklist [38] provided in S3 File. The PainSMART-strategy is a two-stage intervention. Stage one consists of the administration of an educational film and reflection and reinforcement of the film’s key messages prior to the initial physiotherapy consultation. The educational film is entitled ‘Be PainSMART:er’ and will be hereafter named simply as the film. Stage two involves the initial patient-physiotherapist consultation where the film contents are reflected upon and discussed. For details of usual physiotherapy MSKP management see the information relating to the control group.

#### What

*Stage one*. The film was produced and tested by the PainSMART-research group during 2022. The format and content of the film are based on qualitative interview pilot studies (March-April 2022) with patients seeking primary care physiotherapy for MSKP (*n* = 10) and primary care practitioners (*n* = 9) (physiotherapists, occupational therapists, physicians and nurses) [39, 40]. The results of these two studies were combined with cognitive science theories to produce a design framework for the film [41]. The PainSMART-research group then combined the design framework and the results of the two interview studies with modern pain theories, such as the predictive processing theory, Grand Poobah Pain Theory and the CSM, to generate the film’s manuscript and guide the film’s production [9–13]. The film was produced in the period of June-August 2022 by the media company—Miltton Sweden. The film was then pilot tested (September-November 2022) with patients seeking primary care physiotherapy for MSKP (*n* = 10) and primary care practitioners (*n* = 13; physiotherapists, occupational therapists, physicians and nurses) to ensure its key messages were comprehensible and that the film addressed relevant targets [42, 43]. Following these pilot studies minor edits were made to the film.

The film shows a dialog between a physician and a patient with MSKP. The film is seven minutes long and divided into three sections. Section one (4 minutes 30 secs) presents the idea that MSKP is a complex and necessary biopsychosocial protective system that does not accurately reflect the anatomical state of the body [11]. Section one also provides reassurance that MSKP is very rarely caused by serious pathology [44]. Section two (1 minute 30 secs) provides advice on active coping strategies, such as encouraging exercise and work despite some pain, in an attempt to reconceptualise the commonly held belief that a painful body part needs to be rested [13, 17]. Section three (50 secs) aims to prepare patients for their initial healthcare consultation by encouraging them to reflect on the time when their MSKP first developed and their overall life situation with the aim of facilitating a more biopsychosocial consultation. Following the film the patients will rate eight statements that summarise the film’s key messages as listed in Table 1.

**Table 1:** Film content addressing the illness perception dimensions in the Common-sense model of self-regulation (CSM) [12].

CSM dimension	Key messages presented within the film
Identity	• Pain doesn’t necessarily mean damage and pain is a protective system that is necessary and often helpful.
Timeline	• Pain can improve and change over time, irrespective of how long you have had your pain. Many pain problems resolve by themselves, but it can take time.
Consequences	• Pain does not need to stop you from working, exercising or taking part in valued activities. But it can signal that you need to make some adjustments in your life.
Causes	• Pain is very rarely caused by serious pathology. • Pain is not always caused by injury. Pain can also be caused by physical overload, inactivity, imbalance in life or a combination of these factors.
Control	• You can influence your pain through your thoughts and actions but support is available from healthcare professionals. • You can act to improve your pain by staying active, adjusting sleep and diet if necessary.
Emotional representations	• Your thoughts and feelings towards your pain and your mental health influence your pain experience.

The film aims to target patients’ impeding MSKP illness perceptions, improve pain self-efficacy and encourage adaptive self-management strategies, all factors that are hypothesised to improve pain intensity over time. This requires the content of the film to improve factors such as maladaptive perceptions of the causes of MSKP and its persistence (for example, low outcome expectation, anxiety, catastrophising, and fear avoidance beliefs) and low pain self-efficacy. The content of the film addresses all the illness perception dimensions in the CSM and this is outlined in Table 1 [12].

*Stage two*. The consulting physiotherapist will, via four structured questions, initiate a discussion about the film’s contents and the questionnaires the patient has completed prior to the initial consultation. The four questions ask the patient if any of the content within the questionnaires they have completed have generated any thoughts or reflections, whether they had actually seen the film, whether the film generated any thoughts or reflections and if there was anything in particular they took from the film. It is hoped that these questions, in addition to the film providing a shared basis for patient-centered MSKP communication, will facilitate a higher value initial consultation [45, 46].

#### Who and how

The intervention group will be exposed to the film on two occasions. Access to the film is imbedded within the intervention group’s online questionnaires and the film is first made available immediately following completion of baseline background and PROM data collection. Further exposure occurs at the data collection time point prior to the initial physiotherapy consultation. The time from baseline to the initial physiotherapy consultation will vary for each participating patient depending on clinical prioritisation and the accessibility of initial consultations at the participating physiotherapy departments. The film is hosted on RÖ’s Quick channel, viewable on any electronic device and is exclusively available to the intervention group via the questionnaires. In order to reduce the risk of contamination bias the film is not shareable or available via online searching.

All participating physiotherapists will provide initial consultations to patients in both the intervention and control groups. For the intervention group, the initial physiotherapy consultations will take place as usual with the addition of the reflection and discussion around the film and questionnaires. The physiotherapists will not have access to the results of the questionnaires completed by the patients prior to the initial consultation. Any tailoring of the physiotherapists’ responses to the patients’ answers will occur according to the preferences and skills of each individual physiotherapist.

#### Why

**Fig 2:**
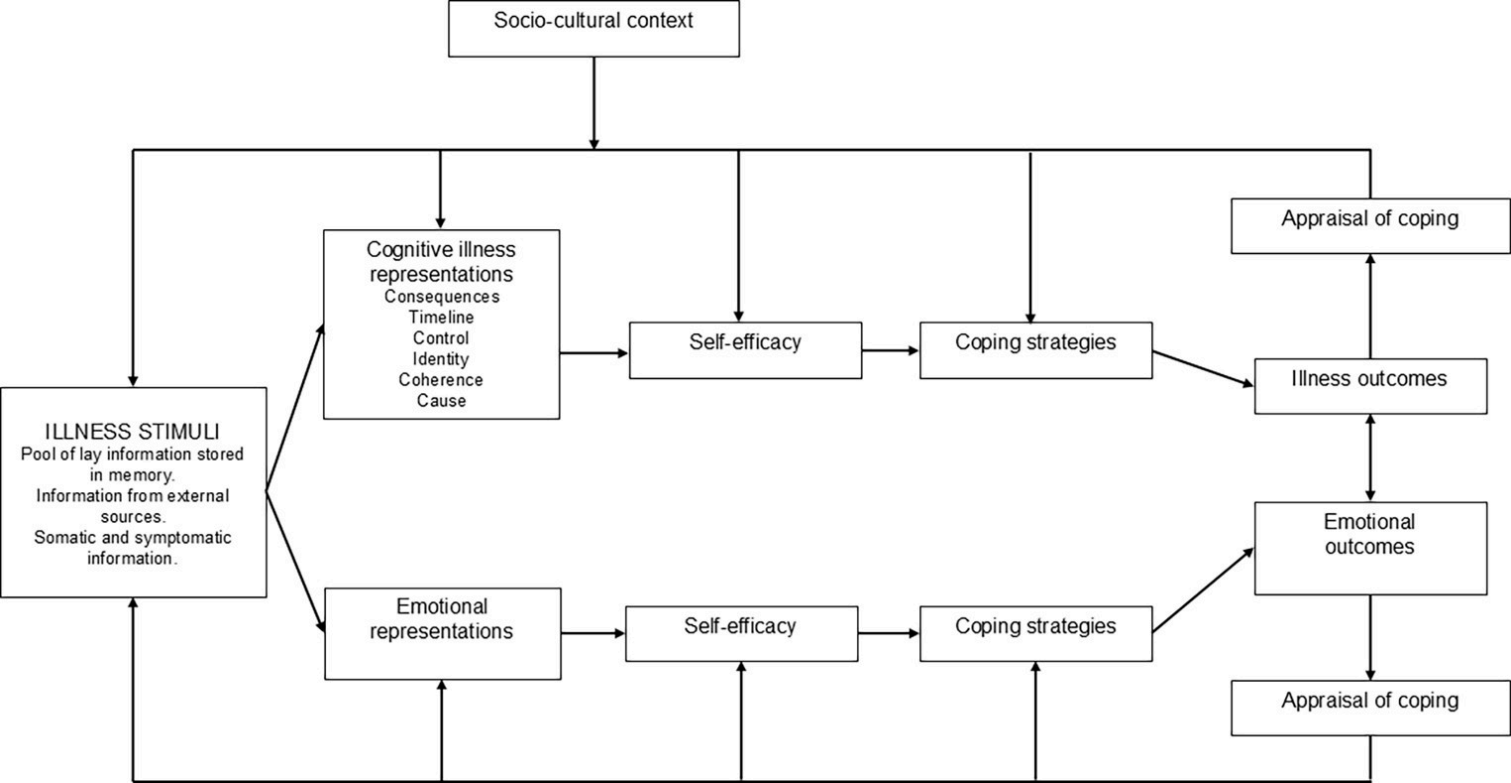
The Common-sense model of self-regulation with integrated self-efficacy concept. The figure is adapted from Fors [50].

*Stage one*. The theoretical rationale for the film’s causal effects can be based on an integration of modern pain theories, the CSM and the concept of self-efficacy [9, 11, 12, 47]. The film and patient’s reflections on the film’s key messages are hypothesised to have effect on patient health outcomes and be mediated by improved cognitive and emotional illness perceptions. The potential direct effects of the film on pain intensity can be linked to factors such as MSKP related concern which directly influence the individual’s perceived threat to bodily integrity [9, 11]. Self-efficacy and illness perceptions have been suggested in respective theoretical models to influence behaviour, including self-management strategies, and thereby affect health outcomes. Individuals´ perceptions about their illness, such as how they perceive its causes and consequences, may influence their self-efficacy. An integration of the CSM and concept of self-efficacy to explain health outcomes has been previously suggested [48, 49]. Fig 2 illustrates such an integration where illness perceptions are hypothesised to influence behaviour and health outcomes directly or indirectly through improved self-efficacy. Therefore, both MSKP illness perceptions and pain self-efficacy could act as mediators of the effects of the PainSMART-strategy. Improvement in an individual’s MSKP illness perceptions and reassurance as to the benign nature of MSKP may improve pain self-efficacy which may in turn change behaviour and affect health outcomes, such as reduced pain intensity, increased levels of physical activity and reduced work absence.

*Stage two*. The rationale for discussing the film at the initial physiotherapy consultation can be based on reinforcing the mechanisms of effect described in stage one, encouraging adherence to the viewing of the film and on the film’s aims to facilitate the initial consultation. All participating physiotherapists will have seen the film prior to the start of patient recruitment, and this enables the physiotherapists to build on and reinforce the film’s key messages (Table 1). As the film is administered prior to the initial physiotherapy consultation it can prepare patients for their consultation and potentially make the patients more central to the process of MSKP-related conceptual change [11]. A discussion between the patient and physiotherapist about the film and aspects salient to the individual patient aims to facilitate a more biopsychosocial consultation, enhance patient participation in their care and improve shared understanding and communication around MSKP [46].

#### Control condition: Usual physiotherapy management alone

Patients first contact their physiotherapy department via telephone or online text-based service and are triaged by a certified physiotherapist. During triage the patients may receive some tailored or generalised advice regarding their presenting condition and possible management strategies or simple exercises. Following triage, the control group will receive online data collection questionnaires identical to those of the intervention group apart from the film and the questions directly related to the film. The initial physiotherapy consultation and continuing physiotherapy management will take place as usual according to the preferences of the patient and physiotherapist with the addition of one question asking the patient to reflect on the questionnaires they have completed as part of the study.

#### Adherence, promotion and monitoring

Patients randomised to the intervention group will rate eight statements about the clarity of the film’s key messages following first exposure (at baseline) and be asked how many times they have viewed the film (out of a maximum of two) as part of the data collection prior to the initial physiotherapy consultation. Patients randomised to the intervention group will be informed that they will discuss the film with the physiotherapist at their initial consultation to improve adherence. All physiotherapists who have conducted at least one of the initial physiotherapy consultations in the RCT will subsequently be asked to complete a questionnaire. This questionnaire asks the physiotherapists to anonymously rate whether they adhered to the study protocol by posing the structured PainSMART-questions to participating patients in the film (four questions) and control (one question) groups respectively. Adherence by the physiotherapist to the protocol regarding the final 30 to 40 consultations (qualitative part) will also be validated via the transcriptions. Non-responders to the questionnaires will receive short messaging service (SMS) and telephone reminders to improve adherence to the interventions.

#### Concomitant care

No limitations will be placed on patients in either group regarding their access to other educational materials, medical advice or treatments during the study. Patients will self-report previous healthcare consultations that they have attended for their MSKP prior to and during the study at baseline, 24–72 hours prior to the initial physiotherapy consultation and at three months post-baseline. Additional information about healthcare visits that have occurred during the study period (baseline to three months) for MKSP complaints will be collected from RÖ’s and RJL’s data registers after the completion of PROM and PREM data collection for all participants.

### Outcomes

The outcomes chosen for this study reflect its aims and theoretical underpinnings. To aid the selection of PROMs and evaluation of PROMs psychometric properties, the COSMIN (COnsensus-based Standards for the selection of health Measurement INstruments) database was searched on the 22nd of February 2023 for systematic reviews evaluating potentially relevant PROMs. Two patient co-designers educated in research methods were also involved in the final choice of study outcome measures in order to incorporate a patient perspective [51].

#### Demographic data

*Patient participants*. Basic demographic data of age, sex, height and weight will be collected at baseline. Key baseline covariate factors, such as pain duration, pain location for the actual presenting complaint, number of other MSKP sites, previous healthcare interventions for the actual presenting complaint the last 3 months, previous healthcare interventions for other MSKP sites the last 3 months, number of co-morbidities, analgesic pain medication use (type and consumption), level of educational, employment including self-reported sick certification/leave for the present MSKP complaint will also be established at baseline.

*Physiotherapist participants*. Basic demographic data of age, sex, department, level of educational and number of years working as a physiotherapist will be collected prior to the start of patient recruitment.

#### Patient Reported Outcome Measures (PROMs)

*Primary outcome measures*. Both primary outcome measures will be collected and analysed as difference between groups in mean aggregate change from baseline and proportion of responders from baseline to 24 hours post-initial physiotherapy consultation and at three months post-baseline. Pain intensity is chosen as a primary outcome for this study as it is a core outcome measure for intervention studies on pain [51, 52]. Average pain intensity, in the previous 24 hours will be measured using a numerical rating scale (NRS) (0–11 from 0 = no pain to 10 = worst imaginable pain) [53]. The average pain intensity NRS rating related to the past 24 hours has been chosen to reduce overlap of the ratings at the separate data collection time points. Previous literature suggests a two-point minimal clinically important difference (MCID) between groups based on the median of study results from systematic reviews of pain intensity ratings in acute and chronic pain [54–56].

The second primary outcome in this study is pain self-efficacy. Pain self-efficacy is defined as “a belief in one’s ability to carry out activities even when in pain” [57]. Pain self-efficacy will be measured using the PSEQ-10 [57]. The PSEQ-10 is a ten-item scale scored as a total (0–60). The PSEQ-10 includes ten statements where participants are asked to rate, from zero to six, how confident they are that they can do certain things despite their pain [57]. The PSEQ is grounded in Bandura’s concept of self-efficacy and has been frequently used in MSKP research [57, 58]. The PSEQ-10 was judged to have good content validity, structural validity, test-retest reliability and responsiveness whilst its internal consistency was judged as excellent in the COSMIN guided systematic review conducted by Dubé et al. [58]. Furthermore, the PSEQ was recommended as the most appropriate PROM for measuring pain self-efficacy in Sleijser-Koehorst et al.’s Delphi-study [59]. The PSEQ-10 has been cross-culturally adapted to Danish in a chronic LBP population and to Swedish for the PSEQ-2 in a MSKP population [60, 61]. The MCID for the PSEQ-10 has been cited to be 5.5–8.5 [58]. The PSEQ-10’s standard error of the mean (SEM) has been cited to range from 1.23 to 5.66 and the minimal detectable change 11.52 [58]. Pain self-efficacy has been chosen as a primary outcome in this study as it has the potential to change rapidly in response to an educational intervention, is thought to mediate the relationship between pain and disability and because higher pain self-efficacy is thought to be protective of the development of chronic MSKP [19–21].

#### Secondary outcome measures

*Secondary outcome PROMs*. Secondary outcome measure PROMs will be collected and analysed as mean aggregate change from baseline to 24–72 hours prior to the initial physiotherapy consultation, 24 hours post-initial physiotherapy consultation and at three months post-baseline. Between group differences in changes over time will also be analysed. NRS pain intensity and pain self-efficacy will be additionally analysed as secondary outcomes for within group and between group differences for data collected at non-primary time points. Average pain intensity, worst pain intensity and best pain intensity in the previous 24 hours will be measured using a NRS (0–11 from 0 = no pain to 10 = worst imaginable pain) [53]. Both primary outcomes measures will be additionally analysed for proportion of responders based on study specific MCID. The MCID for the two primary outcomes will be calculated for interpretation of the within and between-group differences. The MCID will be calculated for the whole cohort using an anchor method [62].

MSKP illness perceptions will be measured using the Brief Illness Perception Questionnaire (BIPQ) [63]. Illness perceptions are defined in this study as “the mental representations and personal ideas that people have about an illness” [64]. The BIPQ was developed based on the CSM to provide a simple and quick assessment of illness representations, emotional representations and illness comprehensibility [64]. The BIPQ contains nine questions, eight use an eleven-point numerical rating scale with anchor statements whilst the final question is a free text question asking participants to list the three most important factors that they believe caused their MSKP [63]. The BIPQ covers the following constructs; cognitive illness representations (consequences, timeline, personal control, treatment control and identity), emotional representations (concerns and emotions), illness comprehensibility and causes [63]. BIPQ will be analysed as a total score (out of 80) according to the scoring instructions from Broadbent et al. [64]. The total score gives an impression of the participant’s perception of the threat or benign nature of their MSKP, with a higher score reflecting a higher threat [64]. For the intervention group only, the BIPQ will be repeated directly after first exposure to the film and questions related to the film’s key messages to assess any immediate change in MSKP illness perceptions. The causal item question will be collected at baseline, directly following first exposure to the intervention, 24–72 hours prior to the initial physiotherapy consultation and again 24 hours after the initial consultation. The BIPQ has been widely used in MSKP research, is validated in Swedish and Norwegian and has shown good concurrent and predictive validity, sensitivity to change and test-retest reliability on meta-analysis [64–66]. MSKP illness perceptions are chosen as a secondary outcome in this study as they have the potential to change rapidly in response to an educational intervention, can mediate the effects of the PainSMART-strategy and as evidence suggests that more negative MSKP illness perceptions are associated with higher pain intensity and poorer physical function [14].

Self-reported level of reassurance of the benign nature of MSKP will be measured using a single reassurance NRS with an eleven-point scale. This question asks the patient how reassured they are that there is not a serious condition causing their MSKP. This question has been adapted from the original research by Sox et al. [67] and has been previously used in research on people with acute LBP [68]. For the intervention group only, the reassurance NRS will be repeated directly after first exposure to the film and questions related to the film’s key messages to assess any immediate change in reassurance. Reassurance is an important measure of the PainSMART-strategy’s effects as the overall level of threat ascribed to a MSKP condition has been linked to pain intensity, disability and pain behaviours [11, 12, 20].

Traditional MSKP coping strategies and psychological flexibility will be measured using the Brief Pain Coping Inventory 2 (BPCI-2) [25]. The BPCI-2 is a 19-item questionnaire, where participants are asked to report on how many days during the last week, they adopted certain pain management strategies (0–7 days). The BPCI-2 contains two sub-scales measuring traditional pain coping strategies and psychological flexibility [25]. Psychological flexibility is defined as “one’s ability to directly and openly contact experiences in the present moment and persisting or changing behaviour according to what the situation affords and one’s personal goals and values” [69]. Higher total (0–133), or subscale scores (0–56 for traditional MSKP coping strategies and 0–77 for psychological flexibility; 4 reverse-scored items) on the BPCI-2 indicate more adaptive coping [25]. The BPCI-2 is based on the Acceptance and Commitment therapy model and has been developed and validated in chronic MSKP populations [25]. The majority of experts who knew of the BPCI-2 in Sleijser-Koehorst et al.’s [59] Delphi study recommended its use. However, the BPCI-2 had not been validated in Swedish or in a primary care population. As such the PainSMART-research group has conducted (May-December 2023) a cross-cultural adaptation and validation of the BPCI-2 in a population of patients seeking primary care physiotherapy for MSKP [70, 71]. Provisional results from this study indicate that the BPCI-2 has acceptable psychometric properties in this population. The BPIC-2 has been chosen as an outcome measure in this study as coping is included in the CSM, passive coping strategies are a risk factor for the development of chronic pain and because psychological flexibility has been found to be significantly associated with pain intensity, physical functioning and psychosocial disability [13, 25].

Self-reported global rating of change will be measured using a single item Global rating of change scale (GRoCs) scored on an eleven-point scale. The eleven-point scale is scored from minus five to plus five, anchored by the terms very much worse (-5), unchanged (0) and completely recovered (+5) in accordance with the recommendation made by Kamper et al. [72]. The score is based on the period from when the patient first contacted the physiotherapy department to the GRoCs data collection time points. GRoCs are widely used in MSKP research and despite being vulnerable to recall bias, have good face and construct validity, test-retest reliability and good sensitivity to change [72]. GRoCs have been recommended as a core outcome measure for MSKP research as they are sensitive to patients’ priorities and are flexible to diverse conditions or pain sites, all factors pertinent to this study [72]. The GRoCs is also included in this study as an anchor for study specific MCID and analysis of the Örebro musculoskeletal pain screening questionnaire´s (ÖMPSQ) predictive ability.

Levels of physical activity will be collected via three self-report screening questions developed for the Swedish national board of health and welfare [73, 74]. These three questions ask the patients how many minutes in the last week they have performed exercise that makes them breathless, how many minutes they have been otherwise physically active besides exercise, for example doing housework or gardening, and how many hours they usually sit during a day (not including sleeping). These questions are included as a secondary outcome measure as the PainSMART-strategy aims to impart the message that maintaining physical activity, even whilst in pain, is important [7]. From these questions it can also be calculated if patients attain a threshold of at least 150 mins per week of moderate intensity physical activity.

*Self-reported analgesic use and sick leave*. Self-reported analgesic medication use (type and level of consumption) for the presenting MSKP complaint will be collected via two questions. The first question establishes analgesic consumption with response options: Yes, on a regular basis; Yes, sometimes; or No. The section question establishes the type of analgesic consumption via six response options: 1. Paracetamol, 2. NSAID, 3. Opioids, 4. Gabapentinoids/Tricyclic antidepressants/Serotonin-norepinephrine reuptake inhibitors, 5. Don’t know, 6. Other. Patients will also self-report if they are currently on sick leave for their presenting MSKP complaint at baseline, 24–72 hours prior to initial physiotherapy consultation, 24 hours post-initial consultation and three months post-baseline. Self-reported current sick leave for the presenting MSKP complaint will be collected via one question with the response alternatives: Yes or No. Self-reported healthcare visits for the presenting MSKP complaint or other MSKP complaints will be collected at baseline relating to the three months prior to baseline. At all other data collection time points, additional healthcare visits for the presenting MSKP complaint will be self-reported. Additional healthcare visits (aside from the study physiotherapist) will be self-reported as yes/no and for which professions the patient has consulted (five options; 1. Physician, 2. Nurse, 3. Another physiotherapist, 4. Chiropractor/Naprapath, 5. Councilor/psychotherapist).

*Healthcare register data*. Data on participating patient’s healthcare consumption, sick leave days, referral for diagnostic imaging and referral to specialist/tertiary care for MSKP during the study period (from baseline data collection to three months) will be collected from RÖ’s and RJL’s healthcare data registers and the national social security database [75] following completion of all PROMs and PREMs data collection. Direct healthcare costs for MSKP per patient during the three-month study period will be collected [76]. A comparison will be made between the intervention and control groups to establish if the PainSMART-strategy can improve health outcomes and the effectiveness of the physiotherapy management pathway.

*Screening tool*. The short form of the ÖMPSQ will be collected a baseline [77]. The ÖMPSQ is a ten-item questionnaire which assesses five constructs; self-perceived function, pain experience, distress, fear-avoidance beliefs and return to work expectancy [77]. The ÖMPSQ was developed in a primary care setting and the questionnaire is scored from 0–100 where a higher score indicates higher risk for future work-related disability [77]. The ÖMPSQ is included in this study to evaluate whether a certain sub-group of patients, based on ÖMPSQ scores, respond to the PainSMART-strategy.

*Patient and Physiotherapist Reported Experience Measures (PREMs)*. In this study PREMs will be assessed after the film for the intervention group only, and 24 hours after the initial physiotherapy consultation for both groups.

- *Evaluation of the intervention group’s experiences of the film*

The intervention group will rate the clarity of the key-messages in the film (Table 1) on a numerical rating scale of zero to ten anchored by the terms, not at all clear and completely clear. These questions are obligatory to increase adherence, reinforce the film’s key-messages and to assess whether the patients receiving the intervention pick up on the film’s intended messages.

- *Evaluation of MSKP-related shared understanding*, *communication*, *participation*, *involvement and emotional support at the initial physiotherapy consultation*

To examine the effects of the PainSMART-strategy on the initial physiotherapy consultation, the patients will answer seven questions, and the physiotherapists will answer three questions in the approximately 24 hours following the initial consultation. Both the physiotherapists and the patients will complete PREMs in order to capture the patient perspective, physiotherapist perspective and to evaluate the interaction, as recommended by Epstein et al. [45]. The PREMs collected in this study are questions adapted from the Swedish National Patient Survey [78]. The National Patient Survey questions are based on validated and reliable instruments and the questions have been adjusted and translated to suit the Swedish healthcare system [78]. The questions the patients will answer cover four dimensions; namely shared understanding of the patients MSKP, participation and involvement, exchange of information and knowledge (communication) and emotional support [78]. The seven questions evaluate if the patients felt that they had the possibility to talk sufficiently about their MSKP, whether they felt included in decision making around their care, whether they had the opportunity to discuss any worries or concerns they had regarding their MSKP and to what extent they discussed what they themselves could do to improve their MSKP and health. The patients will also be asked if they felt they could reach a consensus in understanding with the physiotherapist regarding their MSKP, if they felt the physiotherapist considered their personal MSKP experiences and explained MSKP in a way that they could understand. The physiotherapists in turn will answer three questions rating whether they felt they received sufficient information from the patient to adequately make clinical judgements regarding the patient’s MSKP, whether they and the patient could reach a consensus regarding the patient’s MSKP and whether they felt the patient actively took part in decision making regarding their care. Both the patients’ and physiotherapists’ questions are answered via an eleven-point NRS with anchor statements. A higher score on individual items or total scores indicates a more positive evaluation. These questions have been chosen as they allow evaluation of the patients’ and physiotherapists’ experiences of shared understanding, communication, involvement and support rather than satisfaction as satisfaction levels are known to be biased to patients’ expectations [78]. These questions are included in the study as the dimensions they cover are central to a high-quality consultation [45, 46].

All physiotherapists who have conducted at least one of the approximately 490 initial physiotherapy consultations in the RCT will anonymously rate, on a scale from zero to ten, to what extent they felt that the patients in the film respective control groups were prepared for their initial consultation (i.e. to what extent the patient seemed to have reflected over biopsychosocial factors that may have influenced their pain experience and its impact on function and wellbeing prior to the initial consultation). These follow up questions are aimed at evaluating the consulting physiotherapists’ impression of the impact of the film and questionnaires on the patients’ perceptions of their MSKP and potential contributing factors.

*Qualitative evaluation of the effects of the PainSMART-strategy on communication at the initial physiotherapy consultation*. The PainSMART-strategy is a complex intervention in which the potential mechanisms of effect are diverse [79]. The quantitative part of the RCT will provide limited insight into how the PainSMART-strategy directly influences patient-physiotherapist communication at the initial physiotherapy consultation. Therefore, to further analyse the effects of the PainSMART-strategy on the initial consultation the concluding phase of the RCT will include a qualitative data collection.

**Fig 3:**
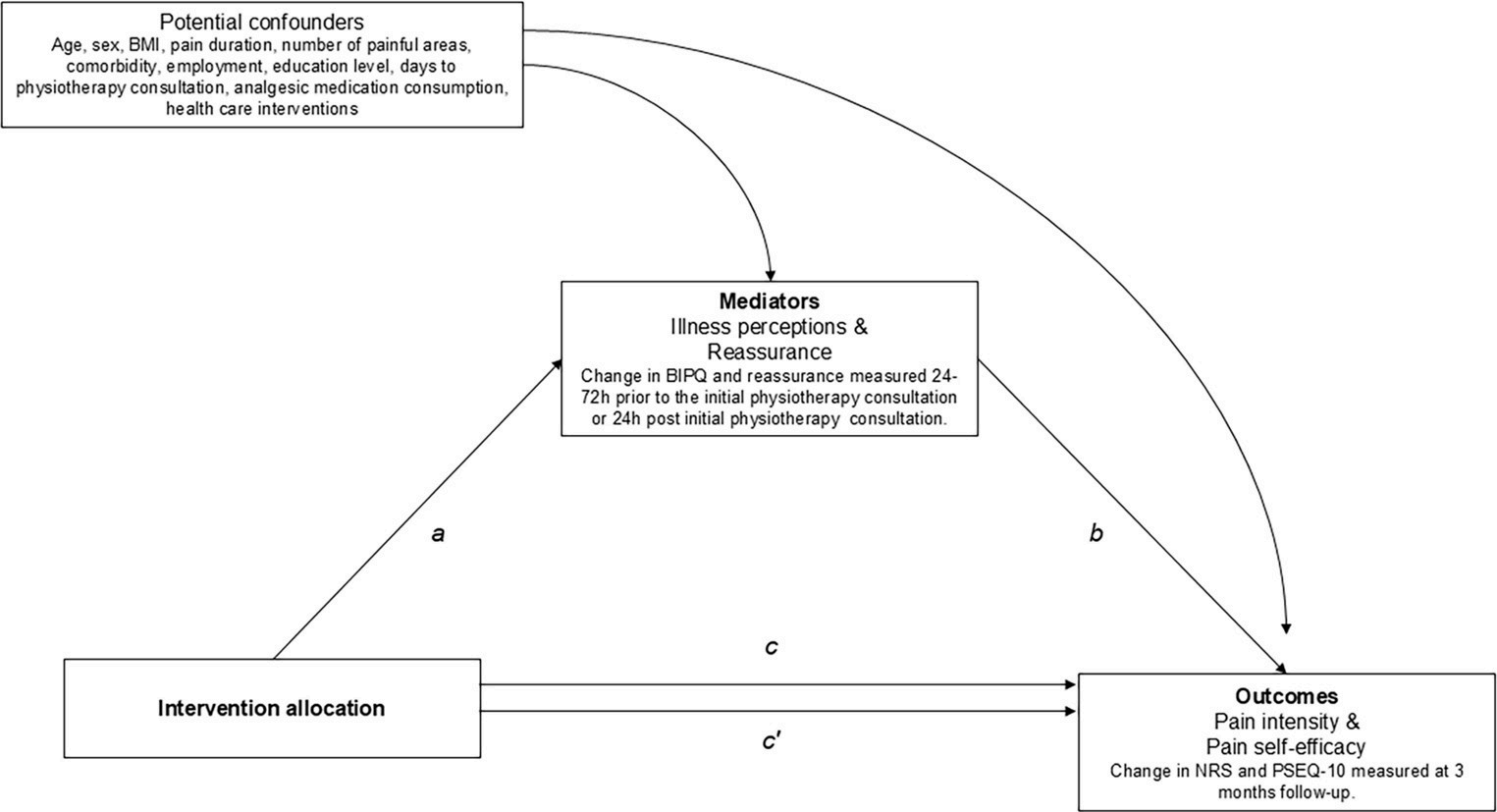
Directed Acyclic Graph (DAG) of the causal pathways for the effect of the PainSMART-strategy. Potential causal effects on the outcomes pain intensity and pain self-efficacy via the hypothesised mediators and the estimated averaged effects adjusted for confounding effects. The potential confounders are measured at baseline. The indirect effect (ab-product) is the average intervention effect through the mediator. a, a-path (the intervention-mediator effect); b, b-path (the mediator-outcome effect); c, c-path (the total effect of the intervention on the outcome, without accounting for potential mediator); c´ (the direct effect of the intervention on the outcome, that works through all other mechanisms excluding the selected potential mediator). BIPQ, Brief Illness Perception Questionnaire; NRS, Numerical Rating Scale; PSEQ-10, Pain Self-Efficacy Questionnaire.

The qualitative phase of the RCT will be based on naturally occurring audio data from the initial physiotherapy consultations of the final 30 to 40 RCT patient participants. All physiotherapists who have conducted at least one of the first 450 initial consultations in the RCT will be asked for additional consent to audio record their consultations with the final group of participating patients. Naturally occurring data has been chosen as the preferred method of data collection for the qualitative phase as it enables an analysis of the PainSMART-strategy that closely mirrors its proposed implementation [80]. This data collection method also enables anonymous data analysis which can reduce the risk of the participating physiotherapists adjusting their consultations in the presence of an observing member of the research team. In addition to the audio recordings these final 30 to 40 patient participants in the RCT will follow the same recruitment strategy, randomisation process (15–20 participants per group) and patient participant timeline for data collection as all other included patient participants (Fig 3). As per protocol, the participating physiotherapists will evaluate the consultation via three questions and subsequently rate to what extent they felt the patient participants were prepared for their initial consultation.

*Patient participant timeline*. Questionnaires will be sent to each patient participant at the following time points:

1. Baseline: the same day as initial triage.
2. 24–72 hours prior to the initial physiotherapy consultation.
3. 24 hours post initial physiotherapy consultation.
4. Three months post-baseline.

For details of what data is collected at each time point see Fig 1.

All patient participants will be enrolled via the standard access pathways to the participating physiotherapy departments in RÖ and RJL. All patient participant data will be collected using the Webropol online questionnaire management service (Linköping, Sweden). For those patients who consent to be contacted by the study coordinators, a SMS-link to the baseline questionnaires will be sent the same day as the initial triage and enrolment (time point *-t*_*1*_). The baseline questionnaires contain more extensive information about the study and the possibility to provide definitive consent. At time point *-t*_*1*_, patients that consent then obtain access to and complete the baseline demographic data and PROMs questionnaires. At time point *t*_*0*_, random group allocation is performed. For patients randomised to the group A intervention, exposure to the film is initiated at time point *t*_*1*_ at the end of the baseline questionnaire. Directly after exposure to the film, questions regarding the film’s key-messages plus a repeat of the BIPQ and reassurance NRS are collected at time point *t*_*2*_. At time point *t*_*3*_, both groups will then receive follow-up PROMs questionnaires approximately 72–24 hours prior to the initial physiotherapy consultation exposure (time point *t*_*4*_) and again at time point *t*_*5*_ which is 24 hours after the initial physiotherapy consultation. PREMs data will also be collected at time point *t*_*5*_. At time point *t*_*6*,_ further exposure to usual physiotherapy management occurs in both groups according to the preferences of the patient and physiotherapist. The final data collection will occur at time point *t*_*7*_, which is three months after each patient’s inception to the study. Patient recruitment initiated 22-01-2024 and is anticipated to be complete 31-12-2024 and all follow-up data collection is anticipated to be complete by 31-03-2025. Results are expected to be published thereafter during 2025 and 2026.

*Physiotherapist data collection*. Physiotherapist consent and background data will be collected via Webropol questionnaires prior to the start of patient recruitment. English language copies of the data collection questionnaires (patient and physiotherapist PROMs and PREMs) are available from the corresponding author on request. The physiotherapists’ evaluations of the initial consultation will be sent to the study coordinators via the messenger function of the electronic journal system within RÖ and RJL. The same function will be used for reminders to physiotherapists if needed to improve response rate. All physiotherapists who have conducted at least one initial PainSMART consultation in the RCT will be sent a follow up Webropol questionnaire containing four questions. These four questions ask the physiotherapists to self-report their adherence to the study protocol for each group and rate their experiences of the participating patients’ degree of preparedness for the initial consultations. For physiotherapists who consent to continue participation in the RCT for the qualitative phase, additional data will be collected via Dictaphone audio recordings of the initial physiotherapy consultation. The audio recordings will then be uploaded to Linköping’s University secure cloud service.

*Sample size*. The sample size calculation for this study is based on its primary hypotheses. The calculations are based on the MCID of 2±SD4 for the NRS for pain intensity and a minimal detectable change of 11.52±SD23.20 for the PSEQ-10 [54–56, 58]. Sample size calculations were computed for both primary outcomes (NRS and PSEQ-10) using a Cohen’s f effect size of = 0.25 (i.e. small-moderate) and a one-sided p-value of p = 0.05 plus a statistical strength of 0.8 (= Power 80%). The largest sample size calculated was for analysis of the NRS (n = 102 per group) and this was adopted as the sample size for this study. To enable mediation and sub-group analyses two subgroups are required. This gives a total sample size of 408 patients. A drop-out rate of approximately 30% was factored into the randomisation sequence giving a total sample of 600 patients. After the first six weeks of patient recruitment (January-February 2024) an internal pilot interim analysis was performed. Of the patient participants who expressed an interest in participating in the study, 65% consented and completed baseline data collection. As of February 29th, 2024, response rates for the questionnaires at time points two and three were approximately 90%. To ensure sufficient follow up data for 408 patients the number of patients to be included at baseline was revised up to 450. This revision, along with the requirement to recruit 30 to 40 patients for the qualitative phase, led to a new total sample size of 490 patients. As a result of the revised sample size an additional randomisation sequence of 200 further patient participants was generated (see allocation).

*Sample size for the qualitative phase*. Inclusion of an additional 30 to 40 patient participants was chosen to increase the probability of achieving saturation of qualitative data and diversity of patient and physiotherapist participants with varying baseline characteristics.

*Recruitment*. Patient participants will be recruited via the five participating primary care physiotherapy departments within RÖ and RJL. To access physiotherapy, patients contact their local rehabilitation or healthcare centre via telephone (TeleQ) or an online text-based service (1177-direkt) and are triaged by a certified physiotherapist. Eligibility for the study will be assessed by the triaging physiotherapist. Potentially eligible patients will be asked via standardised oral or text information for an initial consent to share their contact details with the study’s coordinators. For those patients who consent to be contacted, the study coordinators will send an SMS with a link to the baseline Webropol questionnaire that contains further information about the study and the possibility to provide definitive consent. SMS reminders will be sent to potential participants to encourage recruitment.

One practicing physiotherapist has been appointed as a local PainSMART-champion at each of the participating physiotherapy departments. The PainSMART-champion’s role is to facilitate patient recruitment, communication between the research team and the department and guide study design and implementation. The PainSMART-research group will, along with each PainSMART-champion, provide introductory information about the study prior to the start of patient recruitment. Ongoing communication between the research team and participating departments and their local champions will take place during the patient recruitment period to assist with the study and encourage recruitment. Members of the research team will make further visits to the participating departments as necessary during the study. No incentives are provided to encourage patient recruitment. Recruitment will continue until the sample size is met. Ongoing patient recruitment will be monitored by the study coordinators and is expected to take between six months to one year.

Participating physiotherapists will be all certified physiotherapists employed at the five participating physiotherapy departments. The management team at all five participating departments have consented to the study and encourage all employed physiotherapists to consent to partake in the study.

*Recruitment to the qualitative phase*. Recruitment of new patient participants to the RCT was paused once 450 patient participants had consented and completed baseline data collection (May 21, 2024). The qualitative phase of the RCT will commence once all follow-up data collection has been completed for these 450 participants (October, 2024). Eligible patient participants for the qualitative phase are patients meeting the RCT’s inclusion criteria booked for an initial consultation with a participating physiotherapist. Recruitment will cease once 30 to 40 patients have attended their initial physiotherapy appointment and audio data has been collected.

*Allocation and sequence generation*. All patients who consent to share their contact details with the study coordinators will be randomised. The study coordinators will input all potential patient participants into the code-key system that contains a computerised simple randomisation sequence for 800 patients into group A or B. Patients will therefore be randomly allocated to the intervention or control group based on the consecutive order of their inception to the study. The initial randomisation sequence of 600 patients was generated using SPSS by a blinded statistician prior to the start of patient recruitment (January 2024). Following an internal pilot interim analysis (February 29^th^, 2024) and the decision to add the qualitative phase to the RCT, the total sample size was revised upward to 490 patient participants (from 408). Due to the approximately 65% consent rate at baseline seen during the first month of the RCT, an additional simple randomisation sequence of 200 further patient participants was produced by a blinded statistician using SPSS. This gave a total randomisation sequence of 800 potential patient participants (600 + 200). Once patient participants have consented to the study, the study coordinators document each patient’s participation and group allocation in the electronic journal system for the relevant physiotherapist. This process enables the patients and treating physiotherapists to be concealed from group allocation during the recruitment phase, removing the potential for risk of bias influencing consecutive group allocation.

*Allocation concealment mechanism*. Only patients randomised to the intervention group will be provided access to the film (at time points 1 and 2). Patients randomised to the intervention group will receive the questionnaire battery that includes the film. The control group will receive an identical questionnaire battery aside from the inclusion of the film and questions relating to the film. No mention will be made of the existence of an educational film in the questionnaires or information provided to participants prior to the completion of baseline data collection.

*Blinding*. All participating patients and physiotherapists are blinded to group allocation until after patient consent and baseline data collection. All patients will receive identical information and questionnaires until completion of baseline data collection. Only after completion of baseline data collection will the intervention group receive knowledge of, and access to, the film. The participants in the intervention group are therefore only blinded to the intervention until after the completion of baseline data collection. The participating physiotherapists will only receive knowledge of a patient’s group allocation after the patient has consented to the study. It is not possible to blind for an educational intervention and part of the intervention is aimed at facilitating the initial physiotherapy consultation. This necessitates the physiotherapist having knowledge of whether the patient has been allocated to the intervention or control group. Patients randomised to the control group will be blinded to the existence of an educational film. The study coordinators are not blinded to group allocation but have no role in the patients’ physiotherapy care. Data analysis for all outcomes collected from both study groups will be performed by researchers and an independent statistician blinded to group allocation.

*Data collection and management*. All participant data will be handled and processed by the research team responsible for the study. All participants will be pseudonymised via the use of a code-key system. All patient participant questionnaire data will be collected electronically via the Webropol system. Webropol data is hosted on a secure server within RÖ. Non-responders to the questionnaires will receive SMS and telephone reminders in order to reduce dropouts and missing data. At the conclusion of the study all Webropol data will be transferred to Linköping University’s secure server for analysis. In addition, physiotherapist background data will be collected via Webropol questionnaires and physiotherapy PREMs data via secure messages within RÖ’s and RJL’s electronic journal system. Reminders will be sent to the physiotherapists via the electronic journal system regarding the evaluation of the initial physiotherapy consultations. Physiotherapist initial consultation evaluation data will be stored pseudonymised via code-key on an excel-file and transferred to Linköping University’s server for analysis. The code-keys for patients and physiotherapists will be stored separately from the online data. Dictaphones will be used to record the initial physiotherapy consultations in the qualitative phase of the RCT. The audio files will be uploaded from the Dictaphones to Linköping University’s secure NextCloud service. Patients and physiotherapists will be anonymised during analysis of the audio recordings and transcripts of the recordings. Data management will comply with the European Union’s General Data Protection Regulation (GDPR). The information provided to potential participants will clearly state that their data will be handled and stored securely whilst analysis and reporting of results will be pseudonymised and at group level so that it is not possible to identify individuals. Secure data storage will continue throughout the study and for a minimum of ten years after its conclusion according to current Swedish legislation for research data [81]. Only the research team will have access to the data in the study and will be responsible for data processing together with statistical support staff at Linköping University.

### Statistical methods

This protocol outlines the principal features of the statistical analysis for this study. The attached detailed statistical analysis plan (SAP) was published on Clinicaltrials.gov prior to the completion of patient recruitment to the RCT. The SAP is provided in S4 File.

*Participant characteristics*. ***Patient*.** Group characteristics will be presented as average (mean) values, standard deviations, and frequencies. Baseline analysis between the intervention and control groups will be conducted to ensure the comparability of the groups.

#### Physiotherapist participants

Group characteristics (age, sex, department, highest educational level, number of years of clinical experience, adherence to protocol) will be presented as average (mean) values, standard deviations, and frequencies.

*Analysis of primary outcomes*. Magnitude of within and between-group change on primary outcomes from baseline to 24–72 hours prior to the initial physiotherapy consultation, 24 hours post initial physiotherapy consultation and three months post-baseline will be analysed through mixed models. Data will be compared based on the ‘intention to treat’ (ITT) principle. Sensitivity analyses applying per-protocol, complier average causal effect (CACE) analyses and analyses applying study specific MCID associated with dichotomised anchor response on the GRoC will be explored. Sensitivity analyses to adjust for all measured baseline covariates will also be performed to investigate the presence of equipoise as the result of randomization [82].

*Multiplicity/ type I (α) error*. The outcomes collected in the study are considered as separate entities and, therefore, restrictive multiplicity penalisation of the model is not required [83]. Adjustment will be used for repeated measures over time for separate test conditions.

*Analysis of secondary outcomes*. Magnitude of within and between-group change on secondary PROM outcomes from baseline to 24–72 hours prior to the initial physiotherapy consultation, 24 hours post initial physiotherapy consultation and three months post-baseline will be analysed through mixed models. The causal item question of the BIPQ will be analysed via the grouping of answers into categories, as recommended by Broadbent et al. [64]. Additional analysis will be conducted for the intervention group only for mean aggregate change in the BIPQ (item and total score) and reassurance NRS directly after the first exposure to stage one of the intervention. Data will be compared based on the ‘intention to treat’ (ITT) principle. Sensitivity analyses applying per-protocol, complier average causal effect (CACE) analyses and analyses applying study specific MCID associated with dichotomised anchor response on the GRoC will be explored. Sensitivity analyses to adjust for all measured baseline covariates will also be performed to investigate the presence of equipoise as the result of randomisation [82].

*Analysis of healthcare register outcomes*. Number of healthcare visits, referrals to diagnostic imaging and to specialist/tertiary care, sick leave days and direct healthcare costs from baseline to three months post-baseline will be analysed for both groups and between group differences analysed statistically using t-tests.

*Mediation analyses*. MSKP illness perceptions and level of reassurance as to the benign nature of MSKP are hypothesised, based on an integration of the CSM and concept of self-efficacy (Fig 2), to be potential mediators of the effect of the PainSMART-strategy on pain intensity as well as other secondary outcomes. The integrated model also hypothesises pain self-efficacy to be a mediator in a series of mediators of the PainSMART-strategy’s effects. The effect of MSKP illness perceptions and level of reassurance as to the benign nature of MSKP on pain self-efficacy act as a first step in the causal pathway of the PainSMART-strategy´s effect on health outcomes.

Single causal mediation analysis will be used to analyse indirect effects on pain intensity (NRS) and pain self-efficacy (PSEQ-10) through improvement in MSKP illness perceptions (BIPQ) and reassurance (NRS). The direct acyclic graph model in Fig 3 summarises these causal inferences. Identified potential confounders of the mediator–outcome relationship will be adjusted for in the single mediation analyses. The minimum sufficient adjustment set includes age, sex, BMI, pain duration, number of pain sites, comorbidity, employment, educational level, days to initial physiotherapy consultation, analgesic medicine consumption and previous healthcare interventions. Analyses will also be adjusted for physiotherapist characteristics: age, sex, clinical experience and educational level. The mediation models will be estimated using path-analyses within the framework of Structural Equation Modelling [84]. The results will be reported according to A Guideline for Reporting Mediation Analyses [85].

*Exploratory analyses*. Regression based statistics will be used to explore baseline predictors and mechanisms of longitudinal outcomes. Based on an integration of the CSM and concept of self-efficacy, the effect of MSKP illness perceptions and level of reassurance as to the benign nature of MSKP on pain self-efficacy act as a first step in the causal pathway of the PainSMART-strategy´s effect on health outcomes. As a second step in the causal pathway in the integrated model, the effect on pain self-efficacy is hypothesised to mediate the effects on health outcomes. A secondary objective is to explore if pain self-efficacy mediates the effect of the PainSMART-strategy on health outcomes of interest. The mediation models will be estimated using path-analyses within the framework of Structural Equation Modelling.

*Analysis of patient and physiotherapist experience outcomes (PREMs)*. The intervention group’s scores related to the clarity of the film’s key messages will be presented descriptively. Outcomes relating to the evaluations of the initial physiotherapy consultation will be analysed through descriptive statistics and between group differences for each question. Additional analysis will be conducted for concordance between the patients and physiotherapists ratings on the three paired questions for each group. Physiotherapists’ experiences of their perception of the participating patients’ preparedness for the initial consultation will be analysed through descriptive statistics and between group differences for each question.

*Analysis of physiotherapist adherence*. Data on the physiotherapists’ self-reported adherence to the protocol will be cross-referenced with the film, respective control, group consultations each individual physiotherapist has conducted. This, in combination with the film group patients’ self-reported exposure to the film, will provide data indicating how many of the participating patients received the full PaintSMART-strategy. This data will enable per-protocol and CACE analyses.

*Analysis of qualitative outcomes*. The audio recordings of the initial physiotherapy consultations will be initially screened by a member of the PainSMART-research team. All audio data will be transcribed but information not considered relevant to the qualitative research questions, such as instructions on how to perform a certain exercise, will not be analysed. To ensure anonymous analysis the screening will be conducted by a research team member ignorant to the identity of the participating patients and physiotherapists. Transcription will then be performed by an external party. The transcriptions will be analysed by PainSMART-researchers based on the realist thematic analysis (TA) method described by Wiltshire & Ronkainen [86]. This method has been selected as it enables an empirical analysis, identification and description of the study groups’ understanding and experiences of MSKP [86]. A realist TA approach also enables inferences to be drawn on how unobserved mechanisms may influence the empirically observed experiential themes [86]. This necessitates analysis by experts in MSKP and healthcare communication which, whilst risking a degree of analytical bias, can facilitate development of inferential and dispositional themes that are linked to the RCTs theoretical underpinnings [11, 12, 86]. These themes can in turn aid interpretation of the RCT’s quantitative results. To counter researcher bias, the validity of the experiential themes will be additionally analysed by at least one expert patient research partner involved in the RCT. Further qualitative analyses based on a deductive analysis method will be conducted to explore if the film’s key messages (Table 1) influence the physiotherapy consultation.

*Missing data*. Missing data will be analysed by comparing characteristics (average age, sex) of study participants with and without missing PROM data and analysing the impact of missing data on generalisability [51]. All outcome data will be compared based on the ITT principle. This means that all patients that have been randomised remain in the analysis based on their group allocation. In the event of substantial missing data, evaluation of the mechanisms for missing data will be used [87]. Missing data will otherwise be handled under the missing at random assumption [87]. Multiple Imputation or Maximum Likelihood estimation will be used assuming that missing data is conditional on variables included in the model. Imputation method considering missing at random or not missing at random will be used in the ITT analysis. Patients who cancel their initial physiotherapy consultation will be excluded from the PREM analysis but will remain in the study for PROM and healthcare register data analyses.

*Study monitoring and harms*. There will be no data monitoring committee since the study is independent from the sponsor and the intervention is implemented as an adjunct to usual physiotherapy management with low risk for unexpected adverse events. The PROMs included in the study are sensitive to worsening in the patients’ condition. An internal pilot interim analysis was planned and conducted (February 29^th^, 2024) after approximately 50% of the initially planned sample (n = 229) had consented to the study and reported outcomes at time point two (72–24 hours prior to the initial physiotherapy consultation) and three (24 hours post the initial physiotherapy consultation). This interim analysis enabled evaluation of the viability of the a-priori sample size calculation and the recruitment frequency. The analysis utilised linear mixed models to investigate between group differences (effect size) for change from baseline to time points two and three for outcome. The significance level for the interim analysis was p = 0.05. After interim sample size calculation, the sample size was adjusted slightly to take loss to follow-up into account. The sample size calculation is described under the protocol heading sample size. The trial would have been stopped if the internal pilot interim analysis could not be performed due to not attaining 50% of the sample size at Time point two and three within six months. No further interim analyses are planned.

*Ancillary and post-trial care*. All patient participants will follow the usual physiotherapy care pathway within RÖ and RJL and will therefore have access to other healthcare professions, resources and healthcare levels for additional consultation or management should any unexpected adverse event occur. There will be no restrictions placed on seeking other care during the trial period. Following completion of the study the patients will follow the usual management pathway.

*Ethics*. This study has been approved (25/10/2023) by the Swedish Ethical Review Authority (Etikprövningsmyndigheten), Diary number (Dnr): 2023-05968-01. An amendment to the initial ethical application outlining the concluding qualitative phase of the RCT and follow-up questionnaire for participating physiotherapists was submitted to the Swedish Ethical Review Authority on the July 1^st^, 2024. This amendment was approved on the July 21^st^, 2024, Dnr: 2024-04524-02.

#### Contact details

Swedish Ethical Review Authority, Box 2110, 750 02 Uppsala. Email: registrator@etikprovning.se. Telephone: +46104750800.

*Consent*. ***Participant recruitment*.** All potentially eligible patient participants will receive standardised verbal or text information about the study from the triaging physiotherapist and be asked for consent to share their contact details with the study coordinators. If preliminary consent is obtained, then standardised information will be sent to potential participants via a SMS-link to a Webropol questionnaire. This information contains contact details to the study coordinators and those responsible for the study. Patients who provide written consent will gain immediate access to the baseline questionnaire.

#### Physiotherapist recruitment

All participating physiotherapists will provide definitive consent after receiving verbal information at workplace meetings and further written information in-line with the patient participant recruitment. Consent will be collected via online Webropol questionnaires. Participation in the qualitative phase: all physiotherapists that have consented to the RCT and conducted at least one PainSMART-consultation prior to the start of the qualitative phase will be invited to continue their participation. The eligible physiotherapists will be informed about the qualitative phase by their local PainSMART champion and asked for verbal consent.

*Confidentiality*. The personal details of eligible patients will be shared with the study coordinators via the secure electronic journal system used within RÖ and RJL. All participants (patients and physiotherapists) will receive a unique code-key number to enable pseudonymisation of data and secure data storage within the Webropol system (RÖ) and SPSS program (Linköping’s University). The code-keys for the patient and physiotherapist participants will be stored separately from the secure Webropol (RÖ) server and Linköping’s University data analysis programs. The physiotherapists’ evaluations of the initial consultations will be stored pseudonymised by the use of a code-key. All results will be published at group level. All audio recordings will be deleted from Dictaphones immediately after being uploaded to the NextCloud service. Audio files in NextCloud will be uploaded into a separate file for each physiotherapy department and moved to a central PainSMART-file to remove access to the audio files for all but the research team responsible for the study.

*Declaration of interests*. No conflicting interests. To reduce bias, the study coordinators (RT & MF) will not treat any patients participating in the study in their roles as physiotherapists at the participating departments in Finspång and Linköping.

*Access to data*. Study data will only be accessible to the PainSMART-research group and statistical support team at Linköping’s University. Group level and individual patient data will be available from the research team on reasonable request following completion of the study and publication of the study results.

*Dissemination*. This study protocol has been published via Clinicaltrials.gov to enable public access prior to the inception of patient recruitment. A full statistical analysis plan has been published on clinicaltrials.gov prior to completion of patient recruitment and start of data analysis. The study’s findings will be disseminated and made publicly available in peer-reviewed publications and conference presentations. The study’s results will also be disseminated through regular communication channels within healthcare and university contexts by the research team authorship. If the results of the study are positive the film can be hosted on the 1177 healthcare information platform and be integrated into the primary care physiotherapy MSKP care pathway in RÖ and RJL and even across Sweden.

## Discussion

This research program will determine if the addition of the PainSMART-strategy to usual primary care physiotherapy for MSKP leads to improved PROMs, PREMs and healthcare process outcomes. The project incorporates stringent methodological elements to reduce bias in the clinical trial outcomes, including a-priori protocol registration and sub-study demarcation, clear eligibility criteria, randomization, blinding, as well as a-prior SAP including intention-to-treat, per-protocol, CACE and responder analyses. The incorporation of mixed methods will also highlight factors that may influence outcomes and enable exploration of patient and physiotherapist experiences as well as process outcomes.

This study evaluates a novel approach to educational interventions for patients with MSKP. It is innovative compared to previous studies, as it not only includes a short pre-consultation educational session but also strategies for extending the educational intervention into the consultation and bed for use throughout the patient’s care pathway. For patients who utilise the information, this intervention may enhance pain self-management skills even before healthcare consultation. It may also stimulate reflection, improve communication, and facilitate consensus during the consultation. It is hoped that findings from the research program may fill knowledge gaps in the literature, facilitate patient-practitioner interaction around MSKP and result in improved PROMs, PREMs and process outcomes compared to usual healthcare management.

## Supporting information

S1 File SPIRIT checklist. (PDF)

S2 File WHO trial registration data set. (PDF)

S3 File The TIDieR checklist. (PDF)

S4 File Detailed statistical analysis plan. (PDF)
