# Supplementary material for: The PainSMART project: Protocol for a research program on effectiveness, mechanisms of effect and patient-practitioner experiences of the PainSMART-strategy as an adjunct to usual primary care physiotherapy management for musculoskeletal pain
Source: PLoS One. 2025 Jan 30;20(1):e0316806. doi: 10.1371/journal.pone.0316806 (PMC11781673; doi:10.1371/journal.pone.0316806)
Supplement: S2 File — (PDF) [file pone.0316806.s002.pdf]

Data category	Information
Primary registry and trial identifying number	ClinicalTrials.gov<br>NCT06187428
Date of registration in primary registry	15/12/2023
Prospective Registration:	Yes
Secondary identifying numbers	N/A
Source(s) of monetary or material support	Linköping University, The Research Council of Southeast Sweden (FORSS), Östergötland healthcare region
Primary sponsor	Linköping University
Secondary sponsor(s)	The Research Council of Southeast Sweden (FORSS), Östergötland healthcare region
Contact for public queries	Richard M Thompson, MSc, [+46793345083] [richard.thompson@regionostergotland.se]
Contact for scientific queries	Kajsa Johansson, MSc, PhD<br>Linköping University, Linköping, Sweden
Public title	The PainSMART Research Program: Evaluating a Pain Education Strategy for Patients Seeking Primary Care Physiotherapy PainSMART
Scientific title	The PainSMART Project: A Research Program on Effectiveness, Mechanisms of Effect and Patient-practitioner Experiences of the PainSMART-strategy as an Adjunct to Usual Primary Care Physiotherapy Management for Musculoskeletal Pain
Countries of recruitment	Sweden
Health condition(s) or problem(s) studied	Pain, Musculoskeletal, Pain Acute, Pain Chronic, Patient-Centered Care, Communication, Referral and Consultation, Self Efficacy, Illness Perceptions, Anxiety, Worry, Physical Activity Level, Sickness Absence.
Intervention(s)	Other: PainSMART-strategy<br>Other: Usual physiotherapy management
Key inclusion and exclusion criteria	Inclusion Criteria:<br>- Patients (Both men and women) who, via telephone or online text-based triage, are judged to have benign Musculoskeletal pain (MSKP) and are booked for an initial physiotherapy consultation at one of the five participating physiotherapy departments<br>- Adult patients (18 years or older)<br><br>Exclusion Criteria:<br>- Patients who are judged to require urgent medical examination due to suspected serious pathology (red flags)<br>- Patients who are booked to an initial physiotherapy consultation on the same day as, or the day directly following triage.<br>- Patients referred for physiotherapy following consultation with a tertiary care practitioner (e.g. orthopaedic surgeon, rheumatologist, neurologist)<br>- Patients who cannot communicate in Swedish to the equivalent of a 12-year-old native speaker (as judged by the triaging physiotherapist)<br>- Patients who, through visual impairments, are unable to complete the necessary questionnaires for the study<br>- Patients who are booked for an initial consultation with a physiotherapist who has not consented to taking part in the study
Study type	Interventional
Date of first enrolment	January 22, 2024
Target sample size	490
Recruitment status	Recruiting
Primary outcome(s)	Mean change from baseline and proportion of responders in self-reported Pain Self-efficacy measured using the Pain Self-efficacy questionnaire 10 (PSEQ-10) (within- and between group changes) [Time Frame: The PSEQ-10 will be collected at baseline, 24-72 hours prior to the initial physiotherapy consultation, 24 hours post-initial physiotherapy consultation and again at three months post-baseline]<br>Mean change from baseline and proportion of responders in self-reported Pain Intensity measured using numerical rating scale (NRS) (within- and between group changes) [Time Frame: NRS will be collected at baseline, 24-72 hours prior to the initial physiotherapy consultation, 24 hours post-initial physiotherapy consultation and again at three months post-baseline]
Key secondary outcomes	Secondary Outcome(s)<br>Direct healthcare costs per patient [Time Frame: Total aggregated direct healthcare costs (for all participants in each group) between baseline and three months post-

	baseline]<br>Total number of healthcare consultations attended for musculoskeletal pain for each group (intervention and control) from baseline to three months post-baseline (between group differences) [Time Frame: Total aggregated healthcare consultations attended (for all participants in each group) between baseline and three months post-baseline]<br>Mean change from baseline in self-reported Musculoskeletal pain illness perceptions measured using the Brief Illness Perception Questionnaire (BIPQ) (within- and between group changes) [Time Frame: BIPQ will be collected at baseline, repeated directly after first exposure to the film, 24-72 hours prior to the initial physiotherapy consultation, 24 hours post-initial physiotherapy consultation and again at three months post-baseline]<br>Mean self-reported global rating of change measured using a global rating of change scale (GRoCs) measured at three time points (analysed for both within- and between group changes) [Time Frame: GRoCs will be collected at three time points; first 24-72 hours prior to the initial physiotherapy consultation, 24 hours post-initial physiotherapy consultation and again at three months post-baseline]<br>Mean change from baseline in self-reported traditional musculoskeletal pain coping strategies and psychological flexibility measured using the Brief Pain Coping Inventory 2 (BPCI-2) (within- and between group changes) [Time Frame: BPCI-2 will be collected at baseline, 24-72 hours prior to the initial physiotherapy consultation, 24 hours post-initial physiotherapy consultation and again at three months post-baseline.]<br>Mean change from baseline in self-reported level of reassurance as to the benign nature of MSKP will be measured using a single numerical rating scale (Reassurance NRS) (within- and between group changes) [Time Frame: Reassurance NRS will be collected at baseline, 24-72 hours prior to the initial physiotherapy consultation, 24 hours post-initial physiotherapy consultation and again at three months post-baseline.]<br>Total days of Sickness absence for each group (intervention and control) from baseline to three months post-baseline (between group difference) [Time Frame: Total aggregated days (for all participants in each group) from baseline to three months post-baseline]<br>Total number of referrals for diagnostic imaging for each group (intervention and control) from baseline to three months post-baseline (between group differences) [Time Frame: Total aggregated referrals (for all participants in each group) made between baseline and three months post-baseline]<br>Change in type and frequency of self-reported analgesic medication use (within and between group changes) [Time Frame: will be collected at baseline, 24-72 hours prior to the initial physiotherapy consultation, 24 hours post-initial physiotherapy consultation and again at three months post-baseline.]<br>Mean change in self-reported levels of physical activity measured using three screening questions developed for the Swedish national board of health and welfare (within- and between group changes) [Time Frame: Physical activity will be collected at baseline, 24-72 hours prior to the initial physiotherapy consultation, 24 hours post-initial physiotherapy consultation and again at three months post-baseline.]<br>Total number of referrals to tertiary/specialist care for musculoskeletal pain for each group (intervention and control) from baseline to three months post-baseline (between group differences) [Time Frame: Total aggregated referrals (for all participants in each group) made between baseline and three months post-baseline]<br>One-off screening at baseline with the Örebro Musculoskeletal pain screening questionnaire [Time Frame: Baseline only]
