# Supplementary material for: The PainSMART project: Protocol for a research program on effectiveness, mechanisms of effect and patient-practitioner experiences of the PainSMART-strategy as an adjunct to usual primary care physiotherapy management for musculoskeletal pain
Source: PLoS One. 2025 Jan 30;20(1):e0316806. doi: 10.1371/journal.pone.0316806 (PMC11781673; doi:10.1371/journal.pone.0316806)
Supplement: S4 File — (PDF) [file pone.0316806.s004.pdf]

Statistical analysis plan (SAP)

The PainSMART Project: Statistical analysis plan for a randomised controlled trial on effectiveness, mechanisms of effect and patient-practitioner experiences of the PainSMART-strategy as an adjunct to usual primary care physiotherapy management for musculoskeletal pain

2024 September 25

ClinicalTrials.gov ID: NCT06187428

Version 1.0

The PainSMART Project: Statistical analysis plan for a randomised controlled trial on effectiveness, mechanisms of effect and patient-practitioner experiences of the PainSMART-strategy as an adjunct to usual primary care physiotherapy management for musculoskeletal pain

**Protocol title**	The PainSMART Project: Protocol for a research program on effectiveness, mechanisms of effect and patient-practitioner experiences of the PainSMART-strategy as an adjunct to usual primary care physiotherapy management for musculoskeletal pain
**Trial registration**	Clinical Trials ID no. NCT06187428
	Ethics application: Dnr 2023-05968-01 & Dnr: 2024 04524-02
**SAP Version**	1.0.
**SAP version date**	September 25 2024
**Protocol version**	Version 1.2. Issue date: September 25 2024

Approval and agreement

Kajsa Johansson, Senior Associate Professor, Department of Health Medicine and Caring Sciences, Department of Physiotherapy, Linköping University, Sweden. Principal investigator & head of the PainSMART-project.

Allan Abbott, Professor, Department of Health Medicine and Caring Sciences, Department of Physiotherapy, Linköping University, Sweden. Principal supervisor the PainSMART-project.

Maria Fors, Post-doctoral student, Department of Activity and Health in Linköping, and Department of Health, Medicine and Caring Sciences, Linköping University, Sweden. Physiotherapist, Rörelse & Hälsa, Linköping, Sweden. Project coordinator and post-doc in the PainSMART project.

Richard Thompson, PhD student, Department of Health Medicine and Caring Sciences, Department of Physiotherapy, Linköping University, Sweden. Specialist physiotherapist, Rehab Finspång, Sweden. Project coordinator PainSMART-project.

Henrik Hedevik, Statistician, Department of Health Medicine and Caring Sciences, Department of Physiotherapy, Linköping University, Sweden. Statistical consultant in the PainSMART project.

Persons writing the SAP

Name: Allan Abbott

Position/Title: Professor

Name: Maria Fors

Position/Title: PhD, Post-doctoral student

Approved by

Name: Kajsa Johansson

Position/Title: Senior Associate Professor

Chief investigator/clinical lead

Name: Kajsa Johansson

Position/Title: Senior Associate Professor

Contents

Introduction 1

Background and rationale *1*
Description of specific objectives or hypotheses *1*

Study Methods 2

Trial design *2*
Randomisation *2*
Framework *2*
Determination of sample size *2*
Adherence and protocol deviations *2*
Statistical analysis population *3*
Trial Population *3*
Screening data 3
Eligibility 4
Recruitment 4
Participant drop-out 5
Demography and baseline characteristics 5

Analysis 7

Time frame for data collection *7*
Outcome definitions *7*
Statistical Principles *14*
Timing and Objectives of Interim and Final Analyses *14*
Interim monitoring and analyses 14
Final analysis 14

Analysis methods 14

Primary outcome analysis *14*
Secondary outcome analysis *15*
Patient reported outcome analyses 15
Healthcare register outcome analyses 16
Patient and physiotherapist experience outcome analyses 16

Sensitivity analyses *16*
Mediation analysis *18*
Secondary exploratory analysis *21*
Intervention fidelity 21
Exploratory mediation analysis 21
Predictors and sub-groups 22

Missing data *22*
Statistical software *23*

Data Management Plan 23
References 24

Introduction

Background and rationale

This statistical analysis plan is part of the PainSMART-project which is a collective suite of studies utilising mixed methods. The PainSMART-project was initiated in 2021 with the aim of improving early musculoskeletal pain (MSKP) management through patient-centered MSKP education based on modern pain science. The PainSMART project's motivation is the high burden of MSKP and the issues encountered within primary care clinical practice associated with the persistence of misconceptions surrounding MSKP. The PainSMART-project has to date conducted pilot-studies within the primary care setting that have led to the development of the seven-minute-long digital educational film entitled 'Be PainSMART:er' (hereafter known simply as 'the film'). The film forms the basis for the implementation of a two-stage PainSMART-strategy which consists of exposure to the film prior to the initial physiotherapy consultation and a discussion based on the film between the patient and the physiotherapist at the initial physiotherapy consultation. The aim of this study is to scientifically evaluate the effects of adding the PainSMART-strategy to the current MSKP primary care physiotherapy care pathway in the Swedish healthcare regions of Östergötland (RÖ) and Jönköping (RJL).

Description of specific objectives or hypotheses

The objective of the PainSMART-project is to evaluate the effects of administering the PainSMART-strategy as an adjunct to usual physiotherapy management compared to usual physiotherapy management alone.

Research hypotheses for confirmatory research questions

1. Exposure to the PainSMART-strategy as an adjunct to usual physiotherapy management improves the following outcomes significantly more than usual physiotherapy management alone for patients with MSKP (* = primary outcomes) (H1)

- Reduction in pain intensity*.
- Higher pain self-efficacy*.
- Lower MSKP illness perceptions.
- Higher levels of reassurance of the benign nature of MSKP.
- More adaptive MSKP coping and psychological flexibility.
- Higher self-reported levels of physical activity.
- More positive global ratings of change.
- Lower number of healthcare visits, referrals for diagnostic imaging and to specialist/tertiary care for MSKP, lower analgesic medication use, fewer sick leave days and lower direct costs.
- More positive and concordant patient and physiotherapist evaluations of MSKP-related shared understanding, communication, participation, involvement and emotional support at the initial physiotherapy consultation.

2. Improvements in MSKP illness perceptions and higher levels of reassurance of the benign nature of MSKP mediate improved pain intensity and pain self-efficacy as a result of exposure to the PainSMART-strategy compared to usual physiotherapy management alone.

The null hypothesis (H0) is that there is no clinically or statistically significant differences between the intervention (film) group and the control group (usual physiotherapy management).

Secondary exploratory research questions

1. What baseline factors are predictive of improved patient outcomes after exposure to the PainSMART-strategy?
2. What baseline factors are predictive of the persistence of MSKP?
3. What type of psychological factors and strategies are associated with patient outcomes after exposure to the PainSMART-strategy?
4. Is pain self-efficacy a potential mediator of the PainSMART-strategy's effect on health outcomes?
5. What are patients and physiotherapists experiences of the PainSMART-strategy?

Study Methods

Trial design

This is a two-armed, parallel group, randomised controlled trial in which the intervention group (PainSMART-strategy + usual physiotherapy management) will be compared to a control group (usual physiotherapy management). Treatment allocation is a 1:1 ratio. The control group and statistician will be blinded. One independent statistician will be blinded for the primary analyses. Another independent statistician will be responsible for database development and linkage of patient and physiotherapist data. The PainSMART-strategy is described in full in the trial protocol.

Randomisation

A computerised randomisation sequence for 800 patients into group A (Intervention group) or B (Control group) has been generated by a blinded statistician using SPSS prior to the start of patient recruitment. Participating patients will be allocated to intervention or control group based on the order of their inception to the study. The randomisation is described in full in the trial protocol.

Framework

The trial's hypotheses apply a superiority testing framework where the PainSMART-strategy is hypothesized to be superior in outcome compared to control.

Determination of Sample size

The sample size calculation is described in full in the trial protocol.

Adherence and protocol deviations

The definition of adherence in the intervention group is viewing of the film and receiving the structured PainSMART-questions at the initial physiotherapy consultation. Adherence to viewing of the film will be assessed through the obligatory patient reported experience measure (PREMs) questions directly after the film and patient-reported number of times viewing the film. Receiving the structured PainSMART-questions will be assessed by physiotherapists´ self-reported adherence to the study protocol. The definition of adherence in the control group is receiving one question from the physiotherapist concerning the

questionnaires that the patient had filled in prior to the initial consultation. This will be assessed by physiotherapists´ self-reported adherence to the study protocol.

Adherence in the intervention group will be presented as descriptive statistics (number and percentage) related to the patients' self-reporting of the number of viewings of the film (out of a maximum of two) as well as physiotherapist reported adherence to the structured PainSMART-questions at the initial physiotherapy consultation. Adherence in the control group will be presented with descriptive statistics regarding the number and percentage of patients who have received the question regarding the questionnaires at first consultation. Patients that deviate from protocol are those that, despite randomisation to the intervention group, have not answered the PREMs questions following initial exposure to the film and/or those who have reported not viewing the film at either data collection time points 1 or 2. Patients deviating from protocol are also those patients in the intervention and control groups who have not received the structured questions according to protocol, as reported by the treating physiotherapist. The number of patients deviating from the protocol will be summarised in each allocation group with details of type of deviation provided.

Minor deviation

- No exposure to the structured PainSMART-questions (intervention group)

  * or question regarding the questionnaires at first consultation (control group)#

  * In case of exposure to the film and actively cancelled physiotherapy consultation

  # In case of exposure to baseline questionnaires and actively cancelled physiotherapy consultation

Major deviation

- No exposure to the film and the PainSMART-questions (Intervention group)

Statistical analysis population

The analysis populations include an intention-to-treat population (ITT) and a per-protocol population for this trial. The ITT population will include all randomised patients according to the treatment they were randomised to receive. ITT is the primary efficacy analysis population for this study. A per-protocol population will be considered for participants who received the intervention protocol with no or minor deviation from the protocol.

Trial Population

Screening data

The total number of eligible participants could not be collected during the study as it was considered too resource intensive and would not be sufficiently reliable. Register data on numbers of first physiotherapy consultations for MSKP at the rehabilitation units during the study recruitment period will be collected. Demographic and baseline characteristics (see "Demography and baseline characteristics") will be collected to enable analysis of the representativeness of the study sample (patient participants).

The following demographic data will be collected and presented to enable analysis of the representativeness of the sample of participating physiotherapists: employer, age, sex, level of education, number of years working as a physiotherapist.

Eligibility

The inclusion and exclusion criteria are reported in the protocol. In the case that participants have initially fulfilled these criteria and been randomly allocated to a group but at a later time point have been found to not fulfil the criteria (e.g. the late diagnosis of a serious medical condition), the number of ineligible patients randomised, if any, will then be reported with reasons for ineligibility.

Recruitment

A CONSORT flow diagram will be used to summarize the number of patients who were:

- Eligible for screening
- Ineligible at screening
- Eligible at screening but not randomised - Declined to participate (n=….)
- Randomised (n=...)
- Number excluded post randomisation (n=…) *Reasons ex. consent withdrawal, late diagnosed serious pathology
- Allocated to intervention (n=...)
    - Received allocated intervention (n=...)
    - No exposure to the film and the PainSMART-questions (major deviation) (n=…)
    - No exposure to the PainSMART-questions (minor deviation) (n=…)*
      *Reason: Physiotherapist adherence to protocol (n=…); Cancelled physiotherapy consultation (n=…)
    - Exposure to the whole PainSMART-strategy (n=….)
    - Did not receive allocated intervention, plus reasons (n=...)
- Allocated to control (n=...)
    - Received allocated intervention (n=...)
    - No exposure to the PainSMART-question (minor deviation) (n=…)
      *Reason: Physiotherapist adherence to protocol (n=…); Cancelled physiotherapy consultation (n=…)
- Lost to follow-up
    - Lost to follow-up intervention group
        - PROM + PREM outcomes
            - Time point 1 (n=...)
            - Time point 2 (n=...)
            - Time point 3 - Primary time point one (n=...)
            - Time point 4 - Primary time point two (n=…)
              Physiotherapists - PREM
    - Lost to follow-up control group
        - PROM + PREM outcomes
            - Time point 1 (n=...)
            - Time point 2 (n=...)
            - Time point 3 - Primary time point one (n=...)
            - Time point 4 - Primary time point two (n=…)

Physiotherapists – PREM

- Analysed (n=...)
    - Analysed intervention group PROMs
        - Time point 1 (n=...)
        - Time point 2 (n=...)
        - Time point 3 - Primary time point one (n=...)
        - Time point 4 - Primary time point two (n=…)
    - Analysed intervention health register outcomes (n=...)
    - Analysed intervention PREM outcomes (n=...)
    - Analysed control group PROMs
        - Time point 1 (n=...)
        - Time point 2 (n=...)
        - Time point 3 - Primary time point one (n=...)
        - Time point 4 - Primary time point two (n=…)
    - Analysed control health register outcomes (n=...)
    - Analysed control PREM outcomes (n=...)

Participant drop-out

The number of patients and physiotherapists actively withdrawing from the trial will be reported, with reasons where available. Consent withdrawal will be classified as "complete – no further follow-up or data collection". Lost to follow-up will be classified as "non-response at follow-up". Timing of withdrawal and lost to follow-up will be presented in CONSORT diagram format, and if needed a table, with numbers and reasons for withdrawal, lost to follow-up and exclusion from analysis given at each stage (intervention delivery, prior to the initial physiotherapy consultation, post physiotherapy consultation, three months post baseline). Details on reasons for withdrawal will be presented descriptively.

Demography and baseline characteristics

Patients will be described with respect to demographical factors and baseline characteristics listed below, separately for the two randomised groups.

- Age
- Sex
- Level of education
- Employment including current sickness certification/leave for present MSKP complaint
- Pain duration
- Pain location for actual presenting complaint
- Number of other MSKP sites
- Body mass index (BMI)
- Previous healthcare interventions for the actual presenting complaint in the last three months
- Previous healthcare interventions for other MSKP sites in the last three months

- Analgesic medication use (type and consumption)
- Number of comorbidities
- Risk for future work-related disability – low or high risk according to score on Örebro musculoskeletal pain screening questionnaire (ÖMPSQ), where >50 is classified as high-risk.
- Average waiting time to initial physiotherapy consultation

Baseline PROM scores

- Pain intensity with numerical rating scales (NRS).
    - Worst pain in the last 24 hours (0-10).
    - Best pain in the last 24 hours (0-10).
    - Average pain in the last 24 hours (0-10).
- Pain self-efficacy - Pain self-efficacy questionnaire (PSEQ-10; max score 60).
- Traditional pain coping - Brief Pain Coping Inventory (BPCI-2) traditional pain coping subscale (max score 56).
- Psychological flexibility in pain - BPCI-2 psychological flexibility subscale (max score 77).
- MSKP illness perceptions - Brief Illness Perception Questionnaire (BIPQ; max score 80).
- Level of concern as to the potential likelihood of MSKP being caused by a serious pathology - Single question rated on a NRS (0-10).
- Self-reported physical activity in the past week based on three questions
    - Moderate to intensive exercise (minutes)
    - Other physical activity beside exercise (minutes)
    - Sitting time (minutes during a regular day)

Physiotherapists will be described with respect to age, sex, level of education and number of years working as a physiotherapist. Data will be presented for the whole cohort of physiotherapists and separately for the physiotherapists providing treatment in the two randomised groups.

Normally distributed continuous data will be summarised by mean, standard deviations (SD). For non-normally distributed continuous data, median and lower, upper quartile range, will be provided unless otherwise stated. Categorical data will be summarised by number and percentages. Tests of statistical significance will be undertaken for screening of imbalance between groups in baseline characteristics.

Analysis

Time frame for data collection

Primary and secondary patient reported outcome measures (PROMs) will be collected at four time points:

1. Baseline: the same day as initial triage.
2. 24 -72 hours prior to the initial physiotherapy consultation.
3. 24 hours post the initial physiotherapy consultation (primary time point one).
4. Three months post-baseline (primary time point two).

In addition, patients´ reported experiences of the film and PROMs will be collected directly after exposure to the film in the intervention group. Secondary physiotherapist and patient reported experience measures (PREM) will be collected at time point 3. Healthcare register data relating to the three month study period for each participating patient will be collected after the completion of PROMs and PREMs data collection for all participants.

Data on the physiotherapists´ adherence to the Pain-SMART strategy will be collected after all the first 450 included participants have had their first physiotherapy consultations (after time point three) approximately eight months after the inclusion of the first participant and prior to the start of patient inclusion to the qualitative phase of the trial. For primary and secondary PROM data the primary time points are defined as 24 hours post initial physiotherapy consultation (primary time point one) and three months post-baseline (primary time point two). The data collection is described in full in the trial protocol.

Outcome definitions

The primary and secondary outcomes are described in full in the trial protocol. Both trial arms have the following outcomes measured:

Primary outcomes

- Pain intensity

Self-reported average pain intensity in the previous 24 hours will be measured using numerical rating scales (NRS). Score range: 0-10 with 0 = no pain and 10 = worst imaginable pain.

- Time Frame: collected at baseline, 24 hours post initial physiotherapy consultation and at three months post-baseline.
- Calculation to derive outcome:
    - Between group mean change over time from baseline to 24 hours post initial physiotherapy consultation (primary time point one)
    - Between group mean change over time from baseline to three months post-baseline (primary time point two)

-Pain self-efficacy

Self-reported pain self-efficacy will be measured using the Pain Self-efficacy Questionnaire 10 (PSEQ-10). The PSEQ-10 is a 10-item questionnaire. Score range: 0-60, a higher score indicating greater pain self-efficacy.

- Time Frame: collected at baseline, 24 hours post initial physiotherapy consultation and at three months post-baseline.

- Calculation to derive outcome:
    - Between group mean change over time from baseline to 24 hours post initial physiotherapy consultation (primary time point one)
    - Between group mean change over time from baseline to three months post-baseline (primary time point two)

Secondary outcomes

Patient reported outcome measures (PROMS)

-Pain intensity

Self-reported average pain intensity, worst pain intensity and best pain intensity in the previous 24 hours will be measured using numerical rating scales (NRS). Score range: 0-10 with 0 = no pain and 10 = worst imaginable pain.

- Time Frame: collected at baseline, 24-72 hours prior to the initial physiotherapy consultation, 24 hours post initial physiotherapy consultation and at three months post-baseline.
- Calculation to derive outcome:
    - Within group mean change in average pain intensity over time from baseline to follow-up three months post-baseline (all time points).
    - Between group mean change in average pain intensity over time from baseline to 24 -72 hours prior to the initial physiotherapy consultation.
    - Within and between group mean change in worst pain intensity total score over time from baseline to follow-up three months post-baseline (all time points).
    - Within and between group mean change in best pain intensity total score over time from baseline to follow-up three months post-baseline (all time points).

-Pain self-efficacy

Self-reported pain self-efficacy will be measured using the Pain Self-efficacy Questionnaire 10 (PSEQ-10). The PSEQ-10 is a 10-item questionnaire. Score range: 0-60, a higher score indicating greater pain self-efficacy.

- Time Frame: collected at baseline, 24 -72 hours prior to the initial physiotherapy consultation, 24 hours post initial physiotherapy consultation and at three months post-baseline.
- Calculation to derive outcome:
    - Within group mean change over time from baseline to follow-up three months post-baseline (all time points).
    - Between group mean change over time from baseline to 24 -72 hours prior to the initial physiotherapy consultation.

-Musculoskeletal pain (MSKP) illness perceptions

MSKP illness perceptions will be measured using the Brief Illness Perception Questionnaire (BIPQ). The BIPQ is a nine-item questionnaire where eight items are scored on a 11-point

NRS with anchor statements (score range 0-80, higher score reflecting higher perceived threat). One item is a free text question regarding believed cause of the MSKP (causal item).

- Time Frame: collected at baseline, 24 -72 hours prior to the initial physiotherapy consultation, 24 hours post initial physiotherapy consultation and at three months post-baseline. In addition, the BIPQ is also collected directly after exposure to the film in the intervention group.
- Calculation to derive outcome:
    - Within and between group mean change in the total score over time from baseline to follow-up three months post-baseline (all time points).

-Reassurance of the benign nature of MSKP

Concern about the possibility of MSKP being caused by a serious pathology will be measured using a single 11-point NRS, higher score indicates greater assurance.

- Time Frame: collected at baseline, 24 -72 hours prior to the initial physiotherapy consultation, 24 hours post initial physiotherapy consultation and at three months post-baseline. In addition, the reassurance question is also collected directly after exposure to the film in the intervention group.
- Calculation to derive outcome:
    - Within and between group mean change in the score over time from baseline to follow-up three months post-baseline (all time points).

-Traditional pain coping strategies

Musculoskeletal pain coping strategies will be measured using the Brief Pain Coping Inventory 2 (BPCI-2) sub-scale measuring traditional pain coping strategies (eight questions). Sub-scale score range 0-56, where a higher score indicates more adaptive coping.

- Time Frame: collected at baseline, 24 -72 hours prior to the initial physiotherapy consultation, 24 hours post initial physiotherapy consultation and at three months post-baseline.
- Calculation to derive outcome:
    - Within and between group mean change in the total subscale over time from baseline to follow-up three months post-baseline (all time points).

-Psychological flexibility

Psychological flexibility in pain will be measured using the Brief Pain Coping Inventory 2 (BPCI-2) sub-scale measuring psychological flexibility (11 questions). Sub-scale score range 0-77, where a higher score indicates greater psychological flexibility.

- Time Frame: collected at baseline, 24 -72 hours prior to the initial physiotherapy consultation, 24 hours post initial physiotherapy consultation and at three months post-baseline.
- Calculation to derive outcome:
    - Within and between group mean change in the total subscale score over time from baseline to follow-up three months post-baseline (all time points).

-Physical activity levels

Physical activity in the past week will be measured using three screening questions developed for the Swedish national board of health and welfare.

1. Self-reported physical activity in the past week regarding moderate to intensive exercise (minutes)
2. Other physical activity beside exercise (minutes)
3. Sitting time (minutes during a regular day)

- Time Frame: collected at baseline, 24 -72 hours prior to the initial physiotherapy consultation, 24 hours post initial physiotherapy consultation and at three months post-baseline.
- Calculation to derive outcome:
    - Within and between group mean change in the scores over time from baseline to follow-up three months post-baseline (all time points). Scores on question regarding "Moderate to intensive exercise" and "Other physical activity beside exercise" will also be converted to activity minutes per week < 150min/w or ≥ 150min/w.

-Global rating of change

Global rating of change will be measured using a single item Global Rating of Change Scale (GRoCs) scored on an 11-point scale from minus five to plus five, anchored by the terms very much worse (minus five), unchanged (zero) and completely recovered (plus five). The score is based on the period from when the patient first contacted the physiotherapy department to initiate triage until the data collection points for the GRoCs.

- Time Frame: collected at 24 -72 hours prior to the initial physiotherapy consultation, 24 hours post initial physiotherapy consultation and at three months post-baseline.
- Calculation to derive outcome:
    - The score will be dichotomized into improved (1-5) and unchanged/worse (-5-0).

-Analgesic medication use

Self-reported analgesic medication use (type and consumption) for the presenting MSKP complaint will be established via two questions. The first question establishes self-reported consumption of analgesics with the answer alternatives: Yes, on a regular basis; Yes, sometimes; or No. The second question establishes the type of analgesics taken for MSKP and is ranked according to the analgesics ladder recommendations from the Östergötland health care region pharmaceutical committee (2024) (1. Paracetamol, 2. NSAID, 3. Opioids/gabapentinoids/Tricyclic antidepressants/Serotonin-norepinephrine reuptake inhibitors).

- Time Frame: collected at baseline, 24 -72 hours prior to the initial physiotherapy consultation, 24 hours post initial physiotherapy consultation and at three months post-baseline.
- Calculation to derive outcome:

- Number and proportion of analgesic consumption and type of analgesics in each group at the data collection time points as well as within and between group differences.

-Sick leave

Self-reported current sick leave for the presenting MSKP complaint is established via one question. Answer alternatives: Yes or No.

- Time Frame: collected at baseline, 24 -72 hours prior to the initial physiotherapy consultation, 24 hours post initial physiotherapy consultation and at three months post-baseline.
- Calculation to derive outcome:
    - Number and proportion of patients currently on sick leave at the data collection time points as well as within and between group differences.

-Healthcare visits

Self-reported visits for the presenting MSKP complaint to healthcare practitioners other than the physiotherapist involved in the trial. Answer alternatives: Yes or No.

- Time Frame: collected at baseline, 24 -72 hours prior to the initial physiotherapy consultation, 24 hours post initial physiotherapy consultation and at three months post-baseline.
- Calculation to derive outcome:
    - Number and proportion of visits to other healthcare providers from 24 -72 hours prior to the initial physiotherapy consultation and all prospective data collection timepoints thereafter, as well as within and between group differences.

Healthcare register data outcomes

-Healthcare visits and referrals

Data will be collected from the regional healthcare databases on:

1. Number of healthcare visits for MSKP
2. Number of referrals for diagnostic imaging
3. Number of referrals to tertiary care

- Time Frame: collected for each participating patient for the time period baseline to three months following completion of data collection for all participants.
- Calculation to derive outcome:
    - Number and proportion of referrals and health care visits from baseline to three month follow-up, as well as between group differences.

-Direct cost per patient

Direct cost per patient will be collected from the Östergötland and Jönköping healthcare regions databases (Rebus).

- Time Frame: collected for each participating patient during the study period after the completion of data collection for all participants.
- Calculation to derive outcome:
  - Health care costs (sek) per patient for the MSKP during the study period will be presented as a group mean and standard deviation. Between group mean difference will be analysed.

-Sick leave days

Data on sick leave days will be collected from the Swedish social security database (Försäkringskassan).

- Time Frame: collected for each participating patient for the time period baseline to three months following completion of data collection for all participants.
- Calculation to derive outcome:
  - Number and proportion of sick leave days from baseline to three months follow-up, as well as between group differences.

Physiotherapist and patient reported experience measure outcomes (PREM)

-Patient reported experiences of the film

The perceived clarity of key messages in the film will be measured via rating the clarity for each message (eight) on a 0-10 NRS by patients in the intervention group. A higher score indicates a clearer message.

- Time Frame: collected directly post first exposure to the film in the intervention group.
- Calculation to derive outcome:
  - Descriptive statistics.

-Patient reported experience of the initial physiotherapy consultation

Patients´ experiences of the initial physiotherapy consultation will be measured using seven questions adapted from the National Patient Survey, Sweden. The questions evaluate if the patients felt that they had the possibility to talk sufficiently about their MSKP, whether they felt included in decision making around their care, whether they had the opportunity to discuss any worries and concerns they had regarding their MSKP and to what extent they discussed what they could do themselves to improve their MSKP and health. The patients are also be asked if they felt the physiotherapist considered their personal MSKP experiences and explained MSKP in a way that they could understand. The questions are answered on a NRS score range: 0-10, where a higher score reflects a more positive experience in relation to each question.

- Time Frame: collected 24 hours post the initial physiotherapy consultation.
- Calculation to derive outcome:
  - Descriptive statistics and between group differences in each question. Three of the questions will be matched for concordance with the physiotherapists´ reported experienced questions.

-Physiotherapist reported experience of the initial physiotherapy consultation

Physiotherapists´ experiences of the initial physiotherapy consultation will be measured using three questions adapted from the National Patient Survey, Sweden. The questions evaluate if they felt that they received sufficient information from the patient to adequately make clinical judgements regarding the patient's MSKP, whether they and the patient could reach a consensus regarding the patients MSKP and whether they felt the patient actively took part in decision making regarding their care. The questions are answered on a NRS score range: 0-10, where a higher score reflects a more positive experience in relation to each question.

- Time Frame: collected 24-48 hours post the initial physiotherapy consultation.
- Calculation to derive outcome:
    - Descriptive statistics and between group differences in each question. The questions will be matched for concordance with the patients' PREM questions.

Other measures

-Physiotherapist reported experience of the initial physiotherapy consultation

Physiotherapists´ experiences as to what extent they perceived that the patients were prepared for the initial physiotherapy consultation (i.e., whether the patient seem to have reflected over biopsychosocial factors that may have influenced their pain experience and its impact on their function and wellbeing) will be asked through one question for each group. The question is answered anonymously via NRS, score range: 0-10, where a higher score reflects a perception of the patient being more prepared.

- Time Frame: collected after all participating physiotherapists had their final study consultation.
- Calculation to derive outcome:
    - Descriptive statistics and between group differences in each question.

-Physiotherapists´ adherence to study protocol

Physiotherapists´ self-reported adherence to the study protocol will be measured using one question asking whether they adhered to the protocol by posing the structured PainSMART-questions to participating patients they met from the intervention group. Similarly, one question asking whether the physiotherapist adhered to the protocol by posing the structured question to patients in the control group will also be collected to evaluate adherence. The questions regarding adherence are answered anonymously. In the second, qualitative phase of the trial, self-reported adherence will be validated against data on adherence collected through audio recorded initial physiotherapy consultations. An independent statistician will analyse the concordance between the self-rated adherence and the objective data on adherence from consultations.

- Time Frame: collected eight months post-trial start and after all participating patients in the first part of the PainSMART-trial have had their initial physiotherapy consultation. Data on physiotherapists´ adherence to protocol in the qualitative phase of the trial will be collected after all participating patients have had their initial physiotherapy consultation.
- Calculation to derive outcome:

- Adherence to study protocol will be presented descriptively in the CONSORT flow chart.

Statistical Principles

Confidence intervals and P values

For one-sided tests, the nominal level of type 1 error (a) will be 0,05 and the confidence level for two-sided confidence intervals (CI) will be 95%. The outcomes collected in the study are considered as separate entities and, therefore, restrictive multiplicity penalization of the model is not required (Dmitrienko & D'Agostino 2013). Adjustment will be made for analyses with repeated measures over time for separate test conditions using Bonferroni correction. In the case that distributional assumptions do not hold, e.g., normality etc, data transformation or non-parametric statistical alternatives will be explored.

Timing and Objectives of Interim and Final Analyses

Interim monitoring and analyses

An internal pilot interim analysis was planned and conducted after 50% of the sample had reported outcomes at time point 2 (pre physiotherapy consultation) and 3 (primary time point one, post initial physiotherapy consultation). This to evaluate the viability of the a-priori sample size calculation and the recruitment frequency. The analysis utilised linear mixed models to investigate between group difference (effect size) for change from baseline to time points 2 and 3 in outcome. The significance level for the interim analysis was p=0.05. After interim sample size calculation, the sample size was slightly adjusted when taking loss to follow-up into account. The sample size calculation is described in the trial protocol. The trial would have been stopped if the internal pilot interim analysis could not be performed due to not attaining 50% of the sample size at Time point 2 and 3 within 6 months. No further interim analyses are planned.

Final analysis

The trial is planned to finish recruitment of new patient participants in Dec 2024 – Jan 2025. The final three-month follow-up data collection will therefore be in Mar - Apr 2025. All outcomes will be analysed collectively.

Analysis methods

Primary outcome analysis

-Pain intensity and Pain self-efficacy

Hypothesis testing for the two primary outcomes *pain intensity* and *pain self-efficacy* (score on average pain NRS in the last 24 hours and PSEQ-10) will be based on analysis of group mean change from baseline. Baseline scores and group mean change in primary outcome scores from baseline to primary time points one and two for the intervention and control groups and the between-group change for these time points will be presented as means and CI. The within and between-group change on primary outcomes from baseline to 24 hours post initial physiotherapy consultation and three months post-baseline will be analysed according to ITT principles using a maximum likelihood approach for mixed models adjusted for a relevant covariance structure. The models will be adjusted for baseline covariates where relevant. Baseline factors that will be analysed for potential covariate adjustment include: age, sex, educational level, employment, pain duration, number of pain sites, comorbidities, days to initial physiotherapy consultation, previous healthcare interventions, ÖMPSQ risk category, self-reported sick leave and analgesic medication consumption.

The study specific minimal clinical important differences (MCID) for primary outcomes at primary time points will be calculated for interpretation of the within and between-group differences. The MCIDs will be calculated for the whole cohort using an anchor method (Revicki et al, 2008). A dichotomised anchor response on the GRoC (-5- 0 = not improved; 1-5 = improved) must at least have a small association with change in the primary outcome baseline to primary time points one and two. This will be applied in a receiver operator characteristic (ROC) curve analysing the area under the curve (AUC), specificity, sensitivity and Youden index to attain an optimal cut off point for the MCIDs. MCIDs will be presented in mean and SD.

Secondary outcome analysis

Patient reported outcome analyses

-Continuous variables

Hypothesis testing for secondary outcomes for *pain intensity, pain self-efficacy, MSKP illness perceptions (BIPQ), reassurance of the benign nature of MSKP, traditional pain coping strategies (BPCI-2, sub-scale for pain coping strategies), psychological flexibility (BPCI-2 sub-scale for psychological flexibility),* and *physical activity levels (moderate to intensive exercise, Other physical activity beside exercise, Sitting time)* will be based on analysis of group mean change from baseline. Baseline group mean scores and group mean change for each secondary outcome score from baseline to all time points for the intervention and control groups and the between-group mean change at all time points will be presented as means and CI. The within and between-group change on outcomes from baseline to 24-72 hours prior to the initial physiotherapy consultation, 24 hours post initial physiotherapy consultation and three months post-baseline will be analysed according to the ITT principle using a maximum likelihood approach for mixed models adjusted for a relevant covariance structure. The models will be adjusted for baseline covariates where relevant. Baseline factors that will be analysed for potential covariate adjustment include: age, sex, educational level, employment, pain duration, number of pain sites, comorbidities, days to initial physiotherapy consultation, previous health care interventions, ÖMPSQ risk category, self-reported sick leave and analgesic medication consumption.

The study specific MCIDs for secondary outcomes at all time points will be calculated for interpretation of the within and between-group differences. The MCIDs will be calculated for the whole cohort using an anchor method (Revicki et al., 2008). A dichotomised anchor response on the GRoC (-5- 0 = not improved; 1-5 = improved) must at least have a small association with change in the secondary outcome from baseline to all time points. This will be applied in a ROC curve analysing the AUC, specificity, sensitivity and Youden index to attain an optimal cut of point for the MCIDs. MCIDs will be presented in mean and SD.

-Categorical variables

Hypothesis testing for secondary dichotomous outcomes for *Global rating of change, fulfilment of physical activity recommendation, analgesic medication consumption, self-reported sick leave* and *self-reported healthcare visits* will be based on analysis on differences in proportions over time using generalised linear mixed models. For the secondary multinominal outcome regarding *type of analgesic medications* (1. Paracetamol, 2. NSAID, 3. Opioids/gabapentinoids/Tricyclic antidepressants/Serotonin-norepinephrine reuptake inhibitors) differences in proportions over time will be analysed using generalised logit mixed models. The numbers and proportions for secondary dichotomous outcomes will be reported at all relevant time points for the intervention and control groups as well as between-group comparisons for all time points presented as odds ratio and CI.

Healthcare register outcome analyses

Hypothesis testing for secondary healthcare register outcomes for *number of healthcare visits, referrals to diagnostic imaging* and to *specialist/tertiary care* and *sick leave days* during the trial period will be based on analysis of group mean change from baseline. Baseline group mean scores and group mean change in each of the secondary outcome scores from baseline to all time points for the intervention and control groups and the between-group mean change to all time points will be presented as means and CI. The within and between-group change on outcomes from baseline to 24-72 hours prior to the initial physiotherapy consultation, 24 hours post initial physiotherapy consultation and three months post-baseline will be analysed according to the ITT principle using a maximum likelihood approach for mixed models adjusted for a relevant covariance structure. The models will be adjusted for baseline covariates where relevant. Baseline factors that will be analysed for potential covariate adjustment include: age, sex, educational level, employment, pain duration, number of pain sites, comorbidities, days to initial physiotherapy consultation, previous health care interventions, ÖMPSQ risk category, self-reported sick leave and analgesic medication consumption.

-Direct costs per patient

Hypothesis testing of between group comparisons for the secondary outcome of direct costs per patient during the study period will be based on independent samples t-tests. Group mean and SD will be reported descriptively and between group difference presented as mean and CI.

Patient and physiotherapist experience outcome analyses

Patients receive seven questions and physiotherapists 3 questions regarding their experience of the initial physiotherapy consultation. Between group mean difference for responses to these questions will be analysed using independent sample t-tests. These analyses will be presented as mean and SD for each group and the between group difference in mean and CI. Three of the patient questions will analysed for concordance with the matched physiotherapists´ questions. Concordance within the intervention group and control group will primarily be analyzed by calculating intraclass correlation coefficient (ICC) with 95% confidence interval on each question as well as all three questions combined. Two-way random effects model will be used for ICC, assuming raters to be randomly selected. The ratings are single measures analyzed for consistency, to measure systematic differences between ratings. Patients and physiotherapists are treated as two independent raters, rating each of the three questions.

Sensitivity analyses

The following are all re-analyses of the primary and secondary outcomes under different conditions:

- Per-protocol analysis
- Complier average causal effect (CACE) analysis
- Responder analysis
- Complete case analysis (in case of >5% missing data)

Per-protocol analysis

Per-protocol analyses for the primary and secondary outcomes will be conducted to assess robustness of the ITT analyses and to further inform decisions regarding superiority. The sample of the patients for the per-protocol analyses will be as follows:

- Intervention group: patients receiving the PainSMART-strategy with no or minor deviation from protocol.
- Control group: patients receiving intervention with no or minor deviation from protocol.

Data on the physiotherapists' self-reported adherence to the protocol will be cross-referenced with the intervention respective control group patient consultations that each physiotherapist has conducted. This, in combination with the intervention group patients' self-reported exposure to the film, will provide data on deviation from protocol.

Baseline factors that will be analysed for potential covariate adjustment include: age, sex, educational level, employment, pain duration, number of pain sites, comorbidities, days to initial physiotherapy consultation, previous health care interventions, ÖMPSQ risk category, self-reported sick leave and analgesic medication consumption. Between-group differences on outcomes from baseline to 24-72 hours prior to the initial physiotherapy consultation, 24 hours post the initial physiotherapy consultation and three months post-baseline will be analysed.

Complier average causal effect (CACE) analysis

CACE estimation will be implemented in a structural equation modeling program (eg Mplus) using a maximum likelihood approach. As displayed in the following Directed Acyclic Graph (DAG) model (Figure 1), the covariate (X = baseline score for observed outcome variable) predicting both the outcome (Y = Follow-up score of observed outcome variable) and compliance status (C = dichotomous adherence latent class variable).

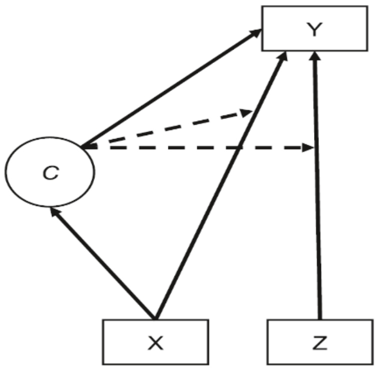

Figure 1. Directed Acyclic Graph (DAG) for CACE analysis

Solid lines from the square boxes for observed variables indicate regressions among these variables. For example, the path from Z (a binary treatment variable) to Y (the outcome) illustrates the treatment effect. The dashed lines originating from the latent class variable C to the regression of observed variables (Z, X) on the outcome suggest that the effects on the outcome can differ across compliance classes. The solid line from the latent class variable to the outcome shows that the means for noncompliers and compliers can differ. The model will be adjusted for other baseline covariates where relevant. Baseline factors that will be analysed for potential covariate adjustment include: age, sex, educational level, employment, pain duration, number of pain sites, comorbidities, days to initial physiotherapy consultation, previous health care interventions, ÖMPSQ risk category, self-reported sick leave and analgesic medication consumption. Between-group differences on outcomes from baseline to 24-72 hours prior to the initial physiotherapy consultation, 24 hours post initial physiotherapy consultation and three months post-baseline will be analysed (Hesser, 2020).

Responder analysis

Responder analyses for the primary and secondary outcomes will be based on calculation of the trial specific MCIDs. The sample of the patients for the responder analyses will be as follows:

- Intervention group:
  - Responders: patients attaining within group mean change equal to and above the study specific threshold for MCID.
  - Non-responders: patients attaining within group mean change below the study specific threshold for MCID.
- Control group:
  - Responders: patients attaining within group mean change equal to and above the study specific threshold for MCID.
  - Non-responders : patients attaining within group mean change below the study specific threshold for MCID.

The group comparison refers to the difference in responder rates between the intervention and the control group using Chi-square test for independence.

Mediation analysis

The planned mediation analysis of the PainSMART-study will help analyse the causal mechanisms of the PainSMART-strategy. If the PainSMART-strategy is found to be effective, the causal mediation analysis will help explain how the strategy works. Conversely, if the strategy is not found to be effective, the causal mediation analysis will help identify where the hypothesised mechanisms broke down.

Primary mediation objective

The primary objective is to confirm if improvement in hypothesised mediators, MSKP illness perceptions and reassurance of the benign nature of MSKP, mediate the effect of the PainSMART-strategy on pain intensity and pain self-efficacy.

Rationale for the hypothesised mediators

MSKP illness perceptions and level of reassurance as to the benign nature of MSKP are hypothesized, based on an integration of the CSM and concept of self-efficacy, to be potential mediators of the effect of the PainSMART-strategy on pain intensity as well as other secondary outcomes. The integrated model also hypothesizes pain self-efficacy to be a mediator in a series of mediators of the PainSMART-strategy's effects. The theoretical rationale underlying the hypothesised mediators in the PainSMART-strategy are explained in full in the trial protocol.

Outcomes

Primary outcomes are average pain intensity in the previous 24 hours measured with a 11-point NRS and pain self-efficacy measured with PSEQ-10. Hypothesised mediators are MSKP illness perceptions measured with the BIPQ and level of reassurance as to the benign nature of MSKP measured with a 11-point NRS.

Data collection

To ensure a temporal sequence between the treatment, mediator, and outcome, data was collected over four sequential time points: 1) baseline, prior to randomisation (primary outcome, mediators, confounders); 2) prior to the initial physiotherapy consultation (mediators); 3) 24 hour post initial consultation (mediators); and 4) three months post baseline (primary outcome).

Mediation models and causal model assumptions

Each of the hypothesised mediator´s potential effect on each primary outcome will be tested in single mediator models. The potential mediating effect of change in the hypothesized mediators at time point 2 (after exposure to the film) and at time point 3 (after exposure to the whole PainSMART-strategy) will be analysed. An overview of the confirmatory mediator models is presented in Table 1. We assume there to be no confounding of the intervention–mediator and intervention–outcome relationships due to random allocation of the intervention (intervention or control group) (Valeri & VanderWeele, 2013). We assume there to be no confounding of the mediator-outcome relationship following adjustment for the sufficient set of potential observed confounders.

Table 1. Overview of confirmatory mediator models

Model	Mediator<br>Pre physiotherapy consultation	Mediator<br>Post physiotherapy consultation	Outcome<br>3 months post baseline
1	Illness perceptions	-	Pain intensity
2	Reassurance of benign nature of MSKP	-	Pain intensity
3	-	Illness perceptions	Pain intensity
4	-	Reassurance of benign nature of MSKP	Pain intensity
5	Illness perceptions	-	Pain self-efficacy
6	Reassurance of benign nature of MSKP	-	Pain self-efficacy
7	-	Illness perceptions	Pain self-efficacy
8	-	Reassurance of benign nature of MSKP	Pain self-efficacy

Confounders

Identified potential confounders of the mediator–outcome relationship will be adjusted for in the single mediation analyses. Potential confounders were identified using the disjunctive cause criterion, which involves selection of measured pre-intervention covariates that are hypothesised to be a cause of the mediator, outcome, or both (VanderWeele, 2019). The

minimum sufficient adjustment set includes age, sex, BMI, pain duration, number of pain sites, comorbidity, employment, level of education, days to initial physiotherapy consultation, analgesic medication consumption and previous health care interventions. The analyses will also be adjusted for physiotherapist characteristics: age, sex, clinical experience and educational level. Baseline measures of the mediator and outcome will also be included in the models.

Statistical analysis

Single causal mediation analysis will be used to analyse the PainSMART´s indirect effects on pain intensity (NRS) and pain self-efficacy (PSEQ-10) through improvement in MSKP illness perceptions (BIPQ) and reassurance of the benign nature of MSKP (NRS). The direct acyclic graph model in Figure 2 summarizes these causal inferences.

For each mediator on each outcome the following will be estimated:

1. The effect for the intervention–mediator relationship, the intervention-outcome relationship and the mediator-outcome relationship.
2. The indirect and direct effects of the intervention on the outcome considering the mediator (single mediated effect).
3. The proportion mediated which is the fraction of the intervention-outcome relationship that is explained by the indirect effect.

Regression-based inference approach will be used for causal mediation analysis. The analysis allows the total effect to be broken down into separate effects (paths) using regression coefficients. The mediation models will be estimated using path-analyses within the framework of Structural Equation Modelling, where all the variables are manifest (i.e., measurable). The mediator models will be constructed with the treatment allocation (binary coded variable 0 = control group, 1 = intervention group) as the independent variable. For each mediator model the intervention-mediator effect (a-path), the mediator-outcome effect (b-path), the average causal mediation effect (indirect effect, ab-product), the average direct effect (c´), and the average total effect (c) will be estimated. The indirect effect is the average intervention effect through the mediator; the direct effect is the average intervention effect that works through all other mechanisms, excluding the selected mediator; and the total effect is the average effect of the intervention on the outcome (MacKinnon, 2012). Effects will be reported with 95% CI.

The mediator models will be adjusted for the baseline score in the dependent variable (outcome), the mediator as well as the potential pre-treatment confounders (MacKinnon, 2012). The interaction term between the intervention allocation and the mediator will be analysed to examine its impact on the indirect effects (MacKinnon et al., 2020). The interaction term will be adjusted for in the outcome model if indicated. The principle of ITT will be followed using full information maximum likelihood in the mediation analyses, if other methods are not indicated.

The results will be reported according to A Guideline for Reporting Mediation Analyses (AGReMA) (Lee et al., 2021).

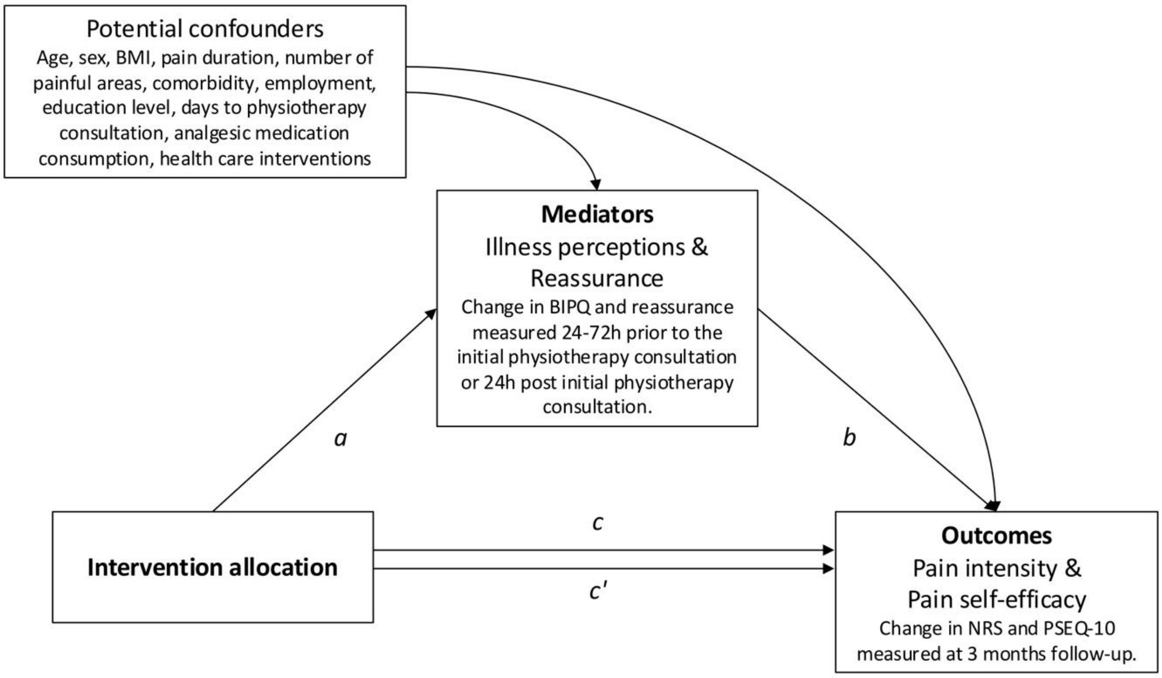

Figure 2. Directed Acyclic Graph (DAG) of the causal pathways for the effect of the PainSMART-strategy on the outcomes pain intensity and pain self-efficacy via the hypothesized mediators and the estimated averaged effects adjusted for confounding effects. The potential confounders are measured at baseline. The indirect effect (ab-product) is the average intervention effect through the mediator. a, a-path (the intervention-mediator effect); b, b-path (the mediator-outcome effect); c, c-path (the total effect of the intervention on the outcome, without accounting for potential mediator); c´ (the direct effect of the intervention on the outcome, that works through all other mechanisms excluding the selected potential mediator). BIPQ, Brief Illness Perception Questionnaire; NRS, Numerical Rating Scale; PSEQ-10, Pain Self-Efficacy Questionnaire.

Secondary exploratory analysis

Intervention fidelity

Physiotherapists´ experiences as to the extent that they perceived the patients as being prepared for the initial physiotherapy consultation will be presented descriptively with means and SDs. The between group difference will be analysed using an independent sample t-test and presented as mean and CI. Analyses will be conducted for the intervention group on group mean change between before and directly after exposure to the film in MSKP illness perceptions (BIPQ item and total score) and on the question regarding reassurance as to the benign nature of MSKP (NRS). Paired sample T-test will be used for analysing the within group change. The results will be presented as means and CI. The intervention group's scores regarding the clarity of the film's key messages will be presented descriptively with means and SD for each question.

Exploratory mediation analysis

The secondary objective is to explore if pain self-efficacy mediates the effect of the PainSMART-strategy on health outcomes of interest. Based on an integration of the CSM and concept of self-efficacy, the effect of MSKP illness perceptions and level of reassurance of the benign nature of MSKP on pain self-efficacy act as a first step in the causal pathway of the PainSMART-strategy's effect on health outcomes. As a second step in the causal pathway in the integrated model, the effect in pain self-efficacy is hypothesized to mediate the effects

on health outcomes. The theoretical rationale underlying the hypothesised mediators in the PainSMART-strategy are explained in full in the trial protocol.

Outcomes and data collection

Outcomes (dependent variables) are health outcomes hypothesised to be improve by the PainSMART-strategy (pain intensity, levels of physical activity, Global rating of change, number of healthcare visits, analgesic medication use, sick leave days). The hypothesised mediator is pain self-efficacy measured with the PSEQ-10. Data collection of the outcomes and mediator is the same as for the primary mediation analyses, see “Mediation analyses”.

Models and causal model assumptions

The potential mediating effects of change in pain self-efficacy at time point 2 (after exposure to the film) and at time point 3 (after exposure to the whole PainSMART-strategy) on the outcome will be analysed. The causal model assumptions are the same as for the primary mediation analyses, see “Mediation analyses”.

Statistical analysis

Causal mediation analysis will be used to analyse the PainSMART’s indirect effects on health outcomes through improvement in pain self-efficacy (PSEQ-10). The mediation models will be estimated using path-analyses within the framework of Structural Equation Modelling.

The relationships that will be estimated for each mediator on each outcome are described in the primary mediation analyses, see “Mediation analyses”. The mediator models will be adjusted for the baseline score in the dependent variable (outcome), the mediator as well as the potential pre-treatment confounders. The interaction term between the intervention allocation and the mediator will be analysed to examine its impact on the indirect effects. The interaction term will be adjusted for in the outcome model if indicated. Missing data will be handled using full information maximum likelihood in the mediation analyses, if other methods are not indicated. The effects will be reported with 95% CI.

Predictors and sub-groups

-Baseline predictive factors

All baseline factors will be explored as potential predictors for health outcomes at time point 4 for the PainSMART intervention group cohort and also for the entire cohort. Regression based statistics will be used. Prediction models will be constructed exploring predictive performance. Internal and external validation of the models will be explored.

-Moderator analyses

Moderator analyses are planned based on the ÖMPSQ risk group categories. The two sub-groups are defined as high risk (score >50) and low risk (score ≤50) for persisting disability and work absence. The dependent variable is the intervention´s effect on health outcomes at time points 3 and 4.

Missing data

Proportion of missing and available data will be investigated through completion rate for each outcome measure and at all data collection time points. The proportion and patterns of missing data in outcomes will be assessed if missing data is >5%. Comparison of characteristics and baseline score on PROMs between participants with and without missing data at one or several data collection time points or according to specific missing data patterns will be analysed to interpret the potential impact of missing data on generalizability.

Imputation will be conducted and handled under the missing at random assumption. In the event of substantial missing data, an evaluation of the mechanisms for missing data will be conducted (Enders, 2011). Multiple Imputation or Restricted Maximum Likelihood estimation will be used assuming that missing data is conditional on variables included in the model. Other imputation methods may be used if necessary.

Statistical software

The primary analyses will be carried out using Statistical Package for Social Sciences (SPSS). Mediation analyses will be performed in Mplus. Other packages, such as R may be used if necessary.

Data Management Plan

Information on data management is provided in the study protocol.

References

Dmitrienko, A., & D'Agostino Sr, R. (2013). Traditional multiplicity adjustment methods in clinical trials. *Statistics in Medicine*, *32*(29), 5172-5218. https://doi.org/10.1002/sim.5990

Enders, C. K. (2011). Analyzing longitudinal data with missing values. *Rehabilitation Psychology*, *56*(4), 267. https://doi.org/10.1037/a0025579

Hesser, H. Estimating causal effects of internet interventions in the context of nonadherence. Internet Interventions 2020, 21, https://doi.org/10.1016/j.invent.2020.100346

Lee, H., Cashin, A. G., Lamb, S. E., Hopewell, S., Vansteelandt, S., VanderWeele, T. J., MacKinnon, D. P., Mansell, G., Collins, G. S., & McAuley, J. H. (2021). A guideline for reporting mediation analyzes of randomized trials and observational studies: the AGReMA statement. JAMA, 326(11), 1045-1056. http://dx.doi:10.1001/jama.2021.14075

Läkemedelskommittén i Östergötland. REK-listan 2024. https://reklista.regionostergotland.se

MacKinnon, D.P. (2012). Introduction to statistical mediation analysis. New York: Routledge.

MacKinnon, D. P., Valente, M. J., & Gonzalez, O. (2020). The correspondence between causal and traditional mediation analysis: The link is the mediator by treatment interaction. Prevention Science, 21, 147-157. https://doi.org/10.1007/s11121-019-01076-4

Revicki, D., Hays, R.D., Cella, D., Sloan, J. (2008). Recommended methods for determining responsiveness and minimally important differences for patient-reported outcomes. Journal of Clinical Epidemiology, 61, 102–109. https://doi.org/10.1016/j.jclinepi.2007.03.012

Valeri, L., & VanderWeele, T. J. (2013). Mediation analysis allowing for exposure–mediator interactions and causal interpretation: theoretical assumptions and implementation with SAS and SPSS macros. Psychological Methods, 18(2), 137. https://psycnet.apa.org/doi/10.1037/a0035596

VanderWeele, T. J. (2019). Principles of confounder selection. European Journal of Epidemiology, 34, 211-219. https://doi.org/10.1007/s10654-019-00494-6
